# Successive waves of COVID 19: confinement effects on virus-prevalence with a mathematical model

**DOI:** 10.1186/s40001-021-00596-6

**Published:** 2021-10-30

**Authors:** S. Abdalla, Duaa Bakhshwin, W. Shirbeeny, Ahmed Bakhshwin, F. Bahabri, Abdulaziz Bakhshwin, Samar M. Alsaggaf

**Affiliations:** 1 Department of Physics, Faculty of Science, King Abdulaziz University Jeddah, P.O. Box 80203, Jeddah, 21589 Saudi Arabia; 2 Department of Pharmacology, Faculty of Medicine, King Abdulaziz University, Jeddah, Kingdom of Saudi Arabia; 3 Department of Pathology, Faculty of Medicine, King Abdulaziz University, Jeddah, Saudi Arabia; 4 Department of Physics, Faculty of Science, Jeddah University, Jeddah, Saudi Arabia; 5 Faculty of Medicine, Alfaisal University, Riyadh, Saudi Arabia; 6 Department of Anatomy, Faculty of Medicine, King Abdulaziz University, Jeddah, Saudi Arabia

**Keywords:** COVID 19, Successive waves, Mathematical model, Confinement effects, Virus-prevalence

## Abstract

**Background:**

A pandemic outbreak of severe acute respiratory syndrome coronavirus 2 (COVID 19) incidence data are largely available online. Until August 17, COVID 19 has hit more than 22 million individuals all over the globe. So, it is urged to get clear information about the prevalence of the virus. Therefore, one can manipulate easily a suitable mathematical model to fit these published data.

**Methods:**

We propose a mathematical model that considers the total population, in 25 countries, either infected by COVID 19 or confined (safe) during the period from November 17, 2019, to August 17, 2020. The model considers the total population as a complex number; the imaginary part is the number of infected individuals and the real part is the number of confined individuals. This classification combined with mathematical treatments leads to a transmission dynamics of the virus to be as wave-like motion. The virus can hit any country either by one wave or by successive waves (up to 11 waves).

**Findings:**

We find net discrimination between the 25 countries investigated in this report. The immediate response to the first attack is a substantial parameter to determine whether the epidemic attack will be in one wave or it can be in successive waves. For example, the best case was such as individuals in China hit by one wave while the individuals in the USA were attacked by nine waves; it is the worst case all over the globe. In addition, the model differentiates between the daily reproduction numbers (*R*_d0_) and the median reproduction number (*R*_0_). We have found that *R*_d0_ decreases exponentially with time from high values down to zero at the wave maximum point; and *R*_0_ varies from a country to another. For example, the virus hit individuals in Germany in *R*_0_ = 1.39 (96% CI 1.01–3.87) and in the USA *R*_0_ = 3.81 (91% CI 1.71–5.15). We have found that twice the virus has hit both the USA and Iran. The great protestation of black matter lives in the USA and the great assemblage of the new Iranian year, on March 21, 2020, have been the cause of the second epidemic attack in both countries.

**Interpretation:**

Our results show that COVID 19 transmission depends on the prompt reaction against the first viral-wave. The reaction depends on both the social behaviour of individuals and on the swift system-decision by the governmental decision-maker(s). The Chinese strictly follow the decision-maker and therefore the virus hit by only one wave; while in the USA, the system-decision was different and the American-responses were different, therefore ten waves followed the first wave.

**Supplementary Information:**

The online version contains supplementary material available at 10.1186/s40001-021-00596-6.

## Introduction

A pandemic outbreak of severe acute respiratory syndrome coronavirus 2 (COVID 19) has lithely spread due to the deficiency of active vaccine and/or effective drugs. Therefore, the confinement scenario is still the most effective tool to resist the spread of COVID 19. It is an unexpected and unprecedented hit and strongly harms the human, economy, health-systems worldwide [1]. The prompt prevalence of the virus combined with the presence of hidden contamination via asymptomatic cases weak the control of the virus. The reproduction number is an effective key to control the viral-progress and to cover its hidden details [2]. We study the confinement effect on the virus prolongation using a mathematical model that accounts for both spread of viral infection and includes time-decaying effects, an individual’s correct knowledge about social distancing. The confirmed COVID 19 infections are more than 22.6 million cases and the deaths exceed 790 thousand worldwide on August 20, 2020 [3, 4].

On the present, and despite the fact that some countries had passed the first epidemic attack, the virus extends to others. For example, countries such as China and Jorden have admitted a firm lockdown state; while others admit free economy positions, the virus hit the first couple by one wave while the others suffered several waves. We believe that the scenario: test-; trace-; isolate-strategy has proved to be effective when combined with good individual protection measures and social distancing [5]. Due to economic reasons, some decision-makers prefer to relax the strict social distancing [6], however, this is not simple because it will be complicated with some extent of pre-symptomatic and asymptomatic infections.

As of June 11, 2020, given the apparent net decrease in the number of infected individuals in some European countries, governments started to move away from the lockdown phase to return to a good level of economic activity. However, on August 15, there are some fears coming from the scientific community about a possible “second” viral epidemic [7]. Here, we present a mathematical model to estimate, to characterize the propagation of the virus, and to predict whether there is another second attack of the virus or not? Using this model, we consider that: (i) the model classifies all individuals into two categories: either confined or infected. (ii) The daily number of coronavirus infections *I* (*t*) occurs in one or successive waves depending on confinement conditions. (iii) The property of causing disease is termed pathogenicity, which is a nonmetric parameter (one cannot measure the pathogenicity). Here, we introduce a measurable parameter that can measure the virus strength as a measurable pathogenic value: “pathogenometric” rate (VSPR) of certain wave: VSPR = [*I*(*t*)/*T*]_max_ where *I*(*t*)_max_ is the maximum number infections of a certain wave, and Tmax is the period necessary to reach *I*(*t*)_max_. The VSPR depends on (but not equals to) the virus pathogenicity. This report proposes a mathematical model to obtain information about the prevalence of COVID 19 virus, in top-infected twenty countries. It reveals that the virus propagates in successive waves. The time delay in responding to the first wave has drastic effects on the prevalence and virus-control. In the present report, we present a mathematical model, which permits one to consider the total population as complex number; its real part is proportional to the confined individuals and the imaginary part represent the infected individuals. The model estimates the reproduction number, and the possible date to reach the maximum point in the wave.

## Methods

### Data sources

To characterize the viral-prevalence dynamics in 25 countries, we fitted a proposed mathematical model to the published data available online [3, 4]. The date of onset and the daily number of new cases in these countries *I*(*t*) in the period from mid-November 17, 2019 until the date of writing the present report august 2020.

### Building the epidemic model

When a directly transmitted infectious disease is present in a certain country with a total population *N*, the number *I*(*t*) of infections represent infected individuals who can move freely. Conversely, *B*(*t*) = C_*B*_ * log [*N*—*I*(*t*)] represents the number of individuals taking measures to resist the virus-propagation while being completely confined. The proportionality-constant, C_*B*_, is chosen to keep a similarity in the magnitude between *I*(*t*) and *B*(*t*) because *B*(*t*) is in the order of million individuals and *I*(*t*) about thousands. Both *B*(*t*) and *I*(*t*) differ in their phase: the model considers *B*(*t*) is completely confined and completely safe, while it considers* I*(*t*) as completely free and completely unsafe (infected). The model classifies the susceptible individuals as infected, *I*(*t*). Mathematically, to represent the phase difference between these two categories, one can write the total population as a complex number:

1 $${N}^{*}\left(t\right) =B\left(t\right)+i\omega t*I\left(t\right),$$

where* B*(*t*) is the real part, and [ωt. *I*(*t*)] is the imaginary part of N^*^(*t*), with $i=\sqrt{-1}$. *ω*, in days^−1^, is defined as the epidemic frequency, which is the ability of individuals to move from the infected phase to the confined phase (see Eq. 6 in “Methods” section). The model considers that the median reproduction number *R*_0_ be greater than one; therefore, *I*(*t*) is assumed to rise exponentially from zero [8, 9]. The period between the first infection and the response to this infection, (*δ*) is important and drastically affects *I*(*t*). In terms of mathematical details, the total population N^*^(*t*) is related to the infected individuals as follows (details in “Methods” section):

2 $${N}^{*}\left(t\right)= { B}_{\infty }+\frac{{B}_{0}{- B}_{\infty }}{1+{[\omega \left(t-\delta \right)]}^{2}}+i\omega \left(t-\delta \right)\left(\frac{{B}_{0}{- B}_{\infty }}{1+{\left[\omega \left(t-\delta \right)\right]}^{2}}\right).$$

The real and imaginary parts of this complex number are given as Real part:

3 $$B\left(t\right)= { B}_{\infty }+\frac{{B}_{0}{- B}_{\infty }}{1+{[\omega \left(t-\delta \right)]}^{2}}.$$

We represent the number of daily infections, *I*(*t*), as the imaginary part:

4 $$I\left(t\right)=\omega \left(t-\delta \right)\left(\frac{{B}_{0}{- B}_{\infty }}{1+{\left[\omega \left(t-\delta \right)\right]}^{2}}\right).$$

We index the rest of the symbols in Additional file 1: Table S1 in "Methods" section.

### The reproduction number (*R*_0_) with successive waves

With the present model, we distinguish between the daily reproduction number, *R*_d0_, and the median reproduction number, *R*_0_ [10, 11]. *R*_d0_ is the daily average number of people infected by one infectious individual; while *R*_0_ is the mean value of all *R*_d0_’s on a certain period starting from zero time until achieving the maximum value of the wave, *T*_max_. To explain this, we consider that the viruses initially attack with high and positive VSPR values; however, the individuals defend by their natural immunities (NIs) [12] and/or any other mitigation conditions. If VSPR is greater than NIs, the slope of the wave will be positive and the viruses will continue their attack. However, if VSPR is lesser than NIs, the slope of the wave will be negative and the viruses will start to decay out and finish their attack. Between these two cases, the wave attains its maximum during a period *T*_max_, where the slope is null. At this condition, the VSPR = NIs, and the number of infections is directly proportional to the mean number of infections that happened from the first infection. In addition, in order to take the exact value of daily reproduction number, one should take into consideration both the negative and positive slopes of the wave. These slopes represent the first derivative of *I*(*t*) that is given by Eq. (4), as following:

5 $$\frac{\partial I\left(t\right)}{\partial t}=\omega \left\{\left(t-\delta \right)\frac{\partial \left(\frac{{B}_{0}{- B}_{\infty }}{1+{\left[\omega \left(t-\delta \right)\right]}^{2}}\right)}{\partial t}+\left(\frac{{B}_{0}{- B}_{\infty }}{1+{[\omega \left(t-\delta \right)]}^{2}}\right)\right\}.$$

Thus, the daily reproduction number R0d is the value obtained by dividing VSPR (*I*_max_/*T*_max_) by the rate given by Eq. (5). One found that the daily reproduction number of infections produced during the infectious period is:

6 $${R}_{\mathrm{d}0}=\frac{\left(\frac{{I}_{max}}{{T}_{max}}\right)}{{\left[\omega \left(t-\delta \right)\frac{\partial \left(\frac{{B}_{0}{- B}_{\infty }}{1+{\left[\omega \left(t-\delta \right)\right]}^{2}}\right)}{\partial t}+\omega \left(\frac{{B}_{0}{- B}_{\infty }}{1+{\left[\omega \left(t-\delta \right)\right]}^{2}}\right)\right]}_{at {T}_{max}}}.$$

Equation (6) gives a daily dimensionless value for *R*_d0_. The average of *R*_d0_ values (*R*_0_) is obtained as:

7 $${R}_{0}=\left(\frac{\sum I(t)}{{T}_{max}}\right).$$

More details about calculating *R*_0_ is found in “Methods” section.

## Results

### Validation of the model—application to daily infections in countries attacked by one wave or more successive waves

**Fig. 1:**
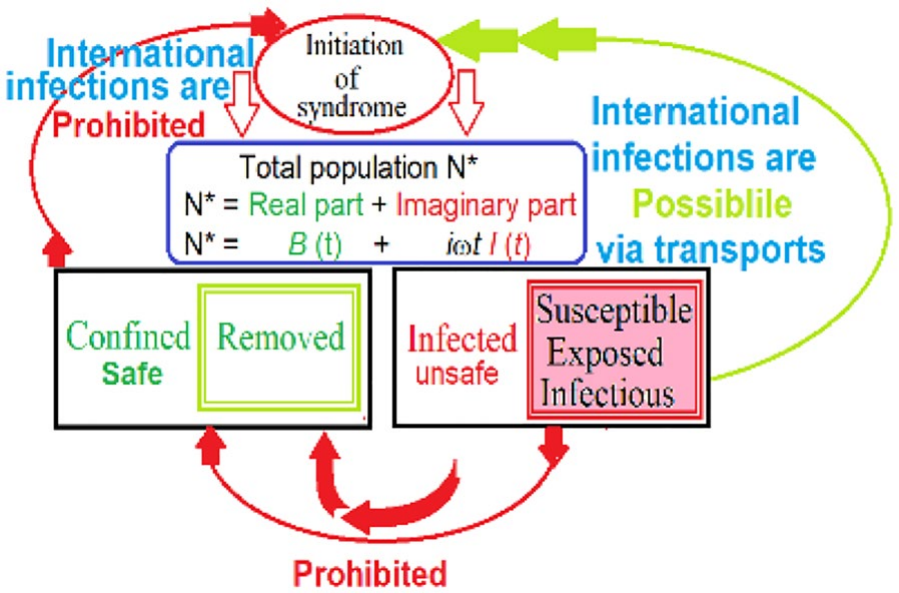
Model building. The model classifies the total population N* into two sections. The first class is totally confined at their domicile and they are completely safe and cannot transmit the virus to the others. The second class is totally infected, free to move, and they are completely unsafe and can transmit the virus to the others. The social, economic, physical, sociological, psychiatric, etc., functions of the two classes are different. Due to the different functions, the model mathematically represents the total population as a complex number (N*). The real part is proportional to the number of confined individuals while the imaginary part represents the number of infected individuals. After the model, the confined class is passive, cannot transmit the virus via international channels; while the infected class is active, and can transmit the virus via internal and international channels. With the present model, the susceptible, the exposed, and the infectious cases are considered as infected cases; while the recovered cases are with the confined cases.

As seen in Fig. 1(A—China), and (B—India), the virus has hit both them in one wave. In these figures, the solid red line represents the calculation values after Eq. (4) while the blue circles stand for the daily published *I*(*t*) values [3, 4]. The calculated data, after equation, (4) fit well the published data that supports the present model. The maximum of the wave in India is |*I*(*t*)|_max India_ = 70,018 infections occur on August 20, 2020. A good fit between calculated and published data stand with the present model. Moreover, we have found a good fit between published and calculated values of *I* (*t*), in more than 20 different countries that support the validity of this model, which are shown in Additional file 1: Figs. S[(A-T)]. In addition, Table 1 reports the time delay (*δ*), the reproduction number *R*_0_, and the VSPR, while the fit parameter-values for these countries are reported in Additional file 1: Table S1 in “Methods” section.

**Table 1:** The virus wave strength as a pathogenometric rate (VSPR)/(*I*/*T*)_max_, the median reproduction number *R*_0_ and the time delay *δ *of 25 countries are reported

Country	VSPR	R(0)		*δ*
USA	438.582	3.81	± 0.31	58
Brazil	447.513	3.89	± 0.56	76
India	347.671	7.51	± 0.61	94
Russia	117.84	9.96	± 1.45	63
S. Africa	108.502	8.44	± 0.69	77
Peru	103.588	7.23	± 1.05	95
Mexico	42.5422	3.84	± 0.31	74
Colombia	67.1729	14.2	± 2.08	72
Spain	150.382	6.34	± 0.52	21
Chili	47.4127	7.01	± 0.87	73
Argentina	308.877	3.35	± 0.68	68
Iran	34.0381	2.14	± 0.18	22
UK	67.9487	3.05	± 0.45	33
S. Arabia	46.4057	6.65	± 0.72	70
Bangladesh	34.5517	2.45	± 0.20	29
Pakistan	59.8624	10.14	± 1.48	57
Italy	130.613	4.01	± 0.59	16
Turkey	171.267	4.67	± 0.38	34
France	113.105	6.56	± 0.54	85
Germany	115.501	1.38	± 0.20	23
Egypt	15.2766	4.50	± 0.49	45
Philippine	28.1045	3.90	± 0.59	73
Indonesia	12.0986	2.61	± 0.19	28
Canada	22.069	1.70	± 0.25	21
Qatar	22.7311	3.97	± 0.33	65
Belgium	25.9077	3.79	± 0.55	29
Norway	13.3285	1.06	± 0.09	7
Netherland	31.0465	5.46	± 0.80	29
China	220.249	1.79	± 0.27	62

**Fig. 2:**
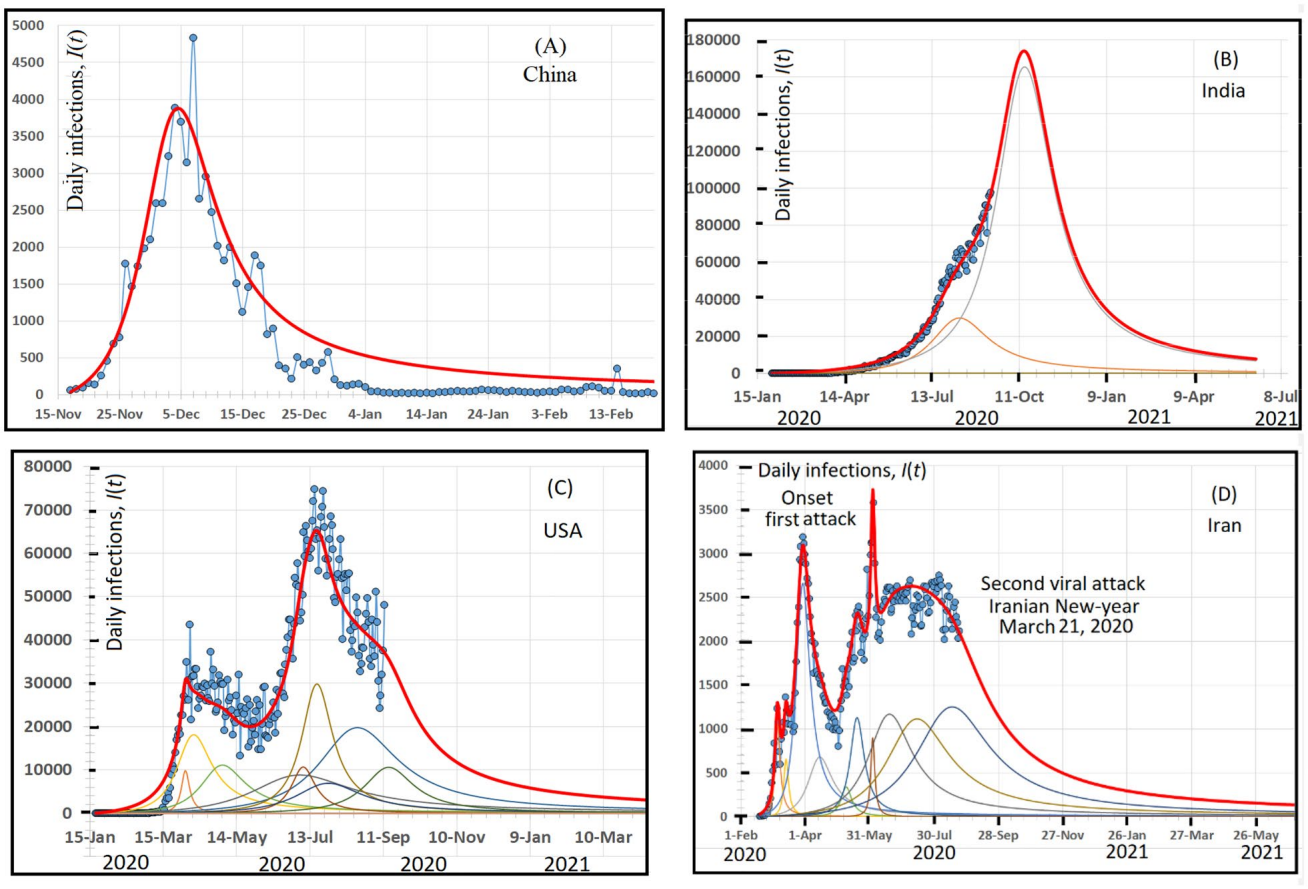
COVID 19 daily transmission in China, India, The USA and Iran fitted during the period from Nov. 17, 2019 up to June 1st, 2021. A cornerstone of the present model is its scientific answer to the question of why does the virus attacks a country more (or less) vigorous with respect to another. For example, the virus has attacked both China (**A**) and India (**B**) in only one wave. Although the enormous population of these countries, one believes that the swift response of the individuals in both countries, in addition to the scientific decision-system of the decision-maker(s) are the reasons that the virus hit by only one wave. While in **C** and **D**, twice-viral attacks have hit the USA and Iran. This is essentially due to (i) the general behaviour of individuals and (ii) the governmental decision-system against virus-prevalence. On June 2020, the USA has some great protestations with 15 million to 26 million people participated in Black Lives Matter. This huge assemblage triggered a second epidemic attack. The same has happened in Iran, on March 21, the Iranians have assembled for the Iranian New Year. The population close contacts were sufficiently high to trigger the second viral attack. In the four figures, the red lines represent the calculated values after Eq. (4) and the blue circles stand for published data. A good fit between the red lines and the blues circle supports the validity of the present model

The time delay (*δ*) and confinement conditions (CCs) vary from a country to another. Increasing *δ* and decreasing social distancing, enhance successive waves to hit any country. For example, Fig. 2C and 2D shows that *I*(*t*) varies as a function of time for the USA (2A) and Iran (2B), respectively. The solid red line represents the calculation values after Eq. (4) while the blue circles stand for the daily published *I*(*t*) values [3, 4]. A good fit between calculated and published data stand with the present model. Figure 2B shows that ten coronavirus waves have attacked Iran in two epidemic attacks; the first attack occurred on February 19, 2020, and had reached a maximum on March 30. A second attack had reached a maximum on June 6, 2020. The reason for these two attacks is the great assemblies for Iranian for the New Iranian Year on March 21, 2020.

### Validation of the model—application to the median reproduction number (*R*_0_)

**Fig. 3:**
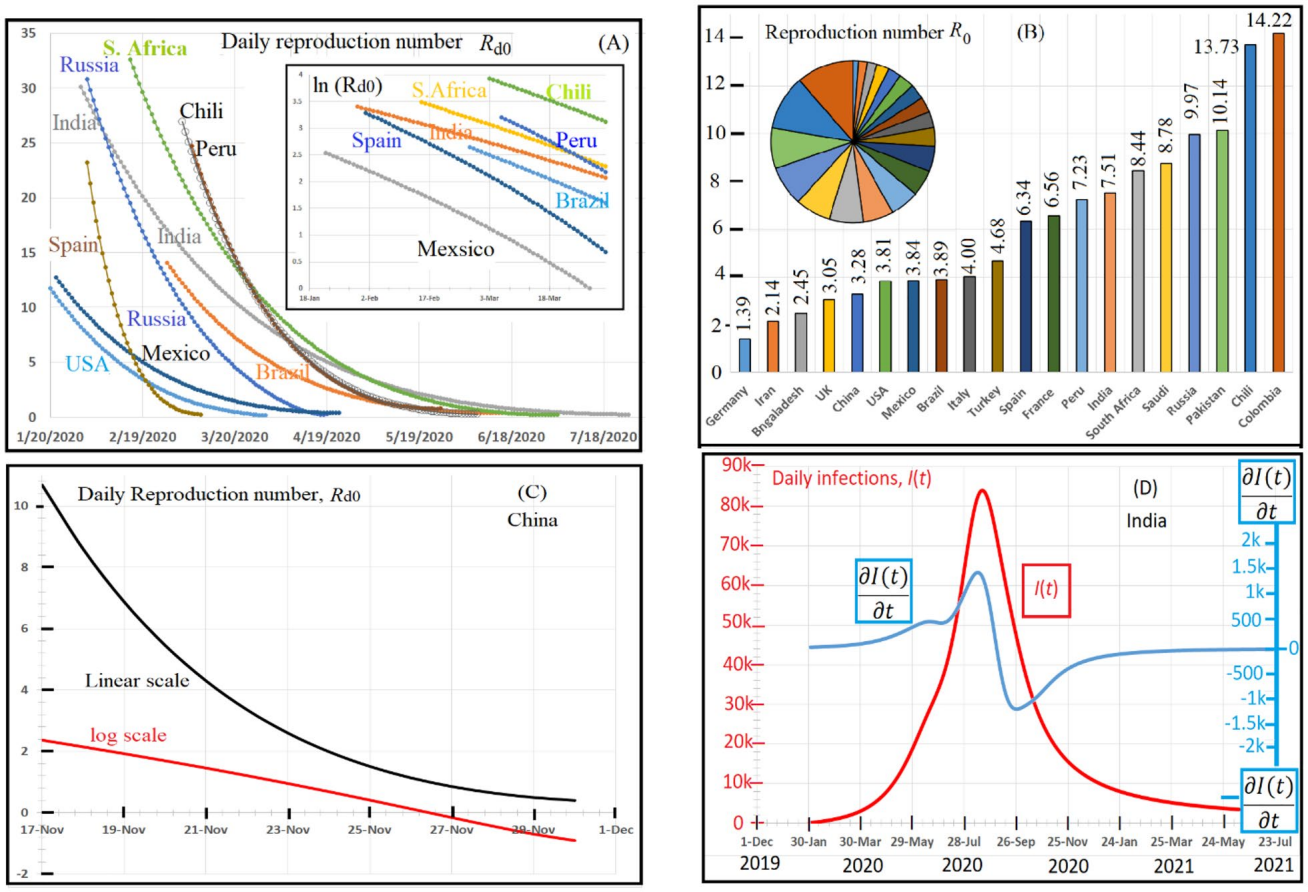
Daily reproduction number variations with time for 11 different countries during the period from Jan 20, 2020, up to July 18, 2020. The daily reproduction number, Rd0, is the number, during certain day, of the new cases infected by an early contagious individual. *R*_d0_ equals [*I*(*t*)/*T*]_max_: *T*_max_ is the time necessary that the wave attains its summit; *I*(*t*)_max_ is the summit value. The median reproduction number *R*_0_ is the mathematical average of *R*_d0_ from onset value down to the minimum value of *R*_d0_. **A** Shows the time variation of *R*_d0_. One notices that *R*_d0_ declines exponentially from high values down to zero. We calculated (after Eq. 6) the daily reproduction number *R*_d0_ in eleven different countries as shown in **A**. As seen, *R*_d0_ decreases exponentially with time from high values (at the onset days) down to minimum values when reaching the maximum point of the wave. At this maximum point, *R*_d0_ tends to zero and infections decline down. The inset in **A** shows the linear behaviour of ln(*R*_d0_) as a function of time that confirms the exponential decrease of *R*_d0_ with time. **B** Shows the median reproduction number *R*_0_ of 20 different countries. We have estimated the mean value of *R*_0_ after Eq. (7). **B** Shows the median reproduction number *R*_0_ for 20 different countries. **C** Confirms the exponential decline of *R*_d0_ as a function of time; the black line represents *R*_d0_ plotted in linear scale while the red line is plotted in logarithmic scale. **D** Shows the first derivative of the daily infection equation (blue line) and the entire wave is plotted in red. One can notice that the summit of the wave corresponds to the inflection point of the blue curve

Figure 3A shows the calculated data, after Eq. (6), of the daily reproduction number *R*_d0_ in nine different countries. As seen, high initial values of *R*_d0_ start at onset time, then, decrease exponentially with time until reaching minimum values, which occur at the maximum point of the wave *T*_max_. The inset in Fig. 3A shows a linear behaviour of ln (*R*_d0_) as a function of time. The median reproduction number *R*_0_, estimated after Eq. 7, of 20 countries is illustrated in Fig. 3B and they are, also, reported in Table 1. *R*_0_ varies in vast range, starting from 1.39 in Germany up to 14.22 in Colombia.

## Discussion

With the present model, we have found that COVID 19 transmits, through certain population, in a successive wave-like prolongation. In general, the virus starts its first attack vigorously with high severity (virus strength). Here, we are in a real need to be more precise and replace the phrase “high severity (virus strength)” by a measurable quantity, which gives a direct value to the virus severity; because the virus pathogenicity is not a measurable one.

In addition, although the production number is an essential parameter to know the rate of viral prolongation and its contagiousness, however, it does not measure the severity of a disease. Therefore, we introduce the term “virus strength pathogenometric” rate (VSPR) of a certain wave as following: $\mathrm{VSPR}={\left(\frac{I(t)}{T}\right)}_{\mathrm{max}}$ where *I*(*t*)_max_ is the maximum value of the wave at time *T*_max_; thus, VSPR is proportional to the viral-severity and it can estimate how the virus is vigorous. This can be explained as following; as the VSPR increases the mitigating factors such as the social distancing and the NIs of the individuals (MFNIs) resist the viral-prevalence in such a way to decrease the VSPR. At the maximum point of the wave, one finds an equilibrium between the VSPR and the MFNIs. After attaining the maximum point, the VSPR decreases down to minimum values. However, the virus will produce several more successive waves if the individuals continue to disregard the MFNIs, in particular the social distancing. We report the values of VSPR for 20 countries in Table 1. These values vary in the range 13.3285 infections day^−1^ (in Norway) < VSPR < 447.513 infections day^−1^ (in Brazil). This vast range of variations of VSPR (VSPR_Brazil_/VSPR _Norway_ is about 33.5) reflects how the individuals behave regarding the social distancing. Thus, VSPR is the maximum number of infection in a certain wave divided by the maximum period to attain the maximum point; It is a rate with a value that estimates a certain proportional average of infections per day; similarly, the first derivative $\frac{\partial I(t)}{\partial t}$ represents a rate with a value proportional to a difference in two successive days infections (Fig. 3D). Therefore, the product of dividing both these rates yields a dimensionless mean of daily infections, ${R}_{0\mathrm{d}}=\frac{VSPR}{\left(\frac{\partial I(t)}{\partial t}\right)}$. Then, one can obtain the median reproduction number, *R*_0_, by simple mathematical averaging of *R*_d0_, ${R}_{0}=\frac{{\sum }_{i=1}^{i=n}{R}_{d0i}}{\left(n\right)}$, where *n* is the number of *R*_d0_ values.

Table 1 shows that *R*_0_ varies in the range 1.07 (in Norway) < *R*_0_ < 13.7 (in Chili). After the considerable variations in *R*_0_ from a country to another, one confirms that COVID 19 has not a constant value of *R*_0_ and it depends on the behaviour of individuals towards the first viral attack. In particular, how they consider the MFNIs, including the social distancing, thus *R*_d0_ drastically depends on MFNIs. The early stage infections (initial escalate of viral infections in a completely susceptible population) leads to high initial values of *R*_d0_, these values varies in the range 10 (China) < *R*_d0_ < about 30 in India, Russia, Peru; then it decreases exponentially down to zero at the maximum point of the wave. These high values of *R*_d0_ have been reported in the early stages of infections in diamond Princess ship [13].

For example, Chinese individuals have kept the infection level constant for a relatively long time, from March 1, 2020 until now, August 21, 2020. With the present model, we can explain this constancy state. Figure 3C illustrates *R*_d0_ as a function of time in the onset period.

As seen, *R*_d0_ decreases exponentially during a relatively short period, from November 17, 2019, until November 27, 2019. Ten days were sufficient to reduce the daily reproduction number from more than ten down to zero. This extraordinary decline of *R*_d0_ is a direct result of the swift reaction of Chinese people to obey the scientific plan of the decision-maker (s) regarding the social distancing. We can confirm the presence of *R*_d0_-exponential decay by the red line in Fig. 3C, which is plotted in natural logarithmic scale while the same data are plotted in linear scale as a black line. One concludes that the Chinese-system of confinement can keep the first wave attack without ongoing other wave attacks. On the contrary, the virus had twice attacked the people in the USA and Iran by two successive epidemic attacks composed of about ten waves as seen in Fig. 2(C-the USA) and (D-Iran). These second epidemic attacks are a direct result of the huge assemblage of the people in both countries. Unfortunately, the virus shows a strong tendency to attack several European countries such as Spain, Germany, Italy, France, UK, etc., as shown in figures (SA—SY), respectively. One can notice that the predicted severity of the second epidemic attack is not as lethal as the first one (it is by far lesser than the premier is). For example, the VSPR values of the second epidemic attack VSPR|_2_ = 27.66 in Germany while VSPR|_1_ = 96.37. One notice that, in Germany, the ratio VSPR|_2_/VSPR|_1_ = 0.29. However, in France, VSPR|_2_/VSPR|_1_ = 46.94/106.71 = 0.44 and in Italy, VSPR|_2_/VSPR|_1_ = 41.58/380.93 = 0.11.

We estimated the fluctuations in *R*_0_ taken into account the fall and rise in daily published-infections. Table 1 shows *R*_0_ combined with the corresponding tolerance.

### Limitations

As all-mathematical infectious disease models, the present one constituently faces some limitations concerning the tolerance of the published data, incomplete counts, unregistered cases, delayed and insufficient tests, etc. Specifically, for the present report, the published data at the onset period are always incomplete, dispersive and unclear. The present model shows that the delay time to respond to the first wave is an essential point and therefore we have paid particular attention to the onset-published data.

It is worth noting that the present model (i) can analyse only a certain type of virus that cannot change its “genetic-imprint”. (ii) The period necessary to attain the maximum number of infections should be equal to or longer than the midpoint of the wave. (iii) The model is in direct opposition to the economical productive factors and did not present a solution to the economical defects of the virus-prevalence. (iv) Contrary to the previous point, the model can search for the optimal condition to find the most effective condition that minimize the confinement factor and maximize the economic conditions.

With the present model, the recovered cases, which they are sensitive to estimate, whether they will return to normal or not? This is not a problem because the recovered cases initially are considered as confined cases. Moreover, Peirlinck et al. [14] have noticed that the undocumented cases of asymptomatic individuals is high, about one-order of magnitude higher than the reported symptomatic cases, which, in addition, highly depends on the testing scenarios of the countries. This is a real limitation for our model. Nevertheless, one should need more tests to well know the real size of the asymptomatic cases and examine if *R*_0_ will alter due to the different infectious period and contact rate.

The decision-makers across the world have differently responded to the COVID 19 pandemic; some of them considered measures, which are highly controversial; others followed an approach towards herd immunity. However, the WHO Director-General has called “herd immunity” as unethical. In fact, suitable control-measures are, extremely, needed to control viral diversity, which increases and, then, different infectious-exposures become ordinary, in particular, when the pandemic attains its highest critical point.

**Fig. 4:**
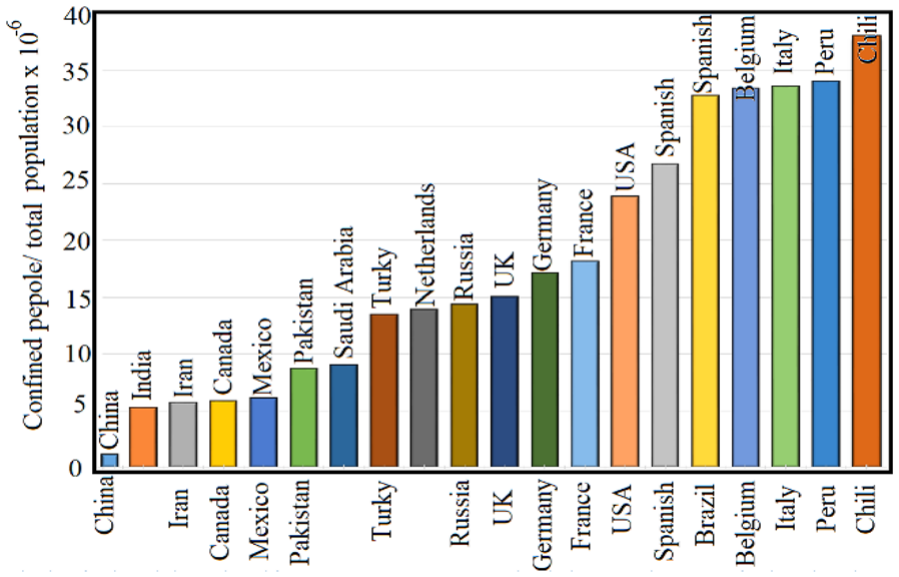
Normalized confined people (to total ones) for 20 countries. One can see that the lower ratio between confined people to the total population is the safest one (China)

Let us compare the number of confined people number *B*(*t*) at a certain time for different countries. In order to get reasonable data, one should take into account several limitations:

(i) One should normalize *B*(*t*) to the total population *N*(*t*) in certain country at certain period.
(ii) *B*(*t*) is a function of time; therefore, one should take the same period for all countries,
(iii) The curve of *B*(*t*) as a function of time has, in most cases, a maximum depending on many factors including the decision-makers response, therefore we will considered the maximum value for each country.
(iv) Figure 4 presents the normalized confined people for different countries.
(v) *B*(*t*) is proportional to the people-safety, regardless the economic effects, *B*(*t*) represents the most suitable parameter for the decision-makers after the given published data, and after Fig. (4). One can conclude that the countries are rated as: (the first is the safest): (1) China, (2) India, (3) Iran, (4) Canada, (5) Mexico, (6) Pakistan, (7) Saudi Arabia, (8) Turkey, (9) Netherlands, (10) Russia, (11) UK, (12) Germany, (13) France, (14) USA, (15) Spain, (16) Brazil, (17) Belgium, (18) Italy, (19) Peru, (20) Chili.
(vi) It is worth noting that the total population (*N*) plays double-contradictory role when calculating the ratio *B*(*t*)/*N*: The first, it decreases, mathematically, the ratio *B*(*t*)/*N*, which increases the possibility to have more saving conditions; while the second it can increases the infections number *I*(*t*) which decreases the possibility to have more saving conditions. In fact, one should carry out more detailed-studies to get an optimum case between these two parameters.

## Summary

We have succeeded to present a scenario depicting the viral prolongation of COVID 19 in one wave or successive waves depending on the swift response of the population towards the onset attack. We found that suitable and good social distancing reduces the median reproduction number from relatively high values at the onset stage (about 10–30) down to zero. The intent of this preliminary work is to introduce the topic of virus-prevalence in successive waves with individual confinement considered as the specific form of mitigation. The model confirms that the confinement is an effective parameter in mitigating the damage of the virus and it shows that the number of infections is drastically affected by the delay in responding to the first wave. In addition, we have found that the second epidemic attack is by far smaller than the onset attack. We have found that the second epidemic attack of the virus. In general, to resist (or even to prevent) epidemic growth, our calculations suggest some lockdowns that be maintained for two months. However, the optimum conditions between health safety and economic damage should be achieved to get the best lockdown timing.

## Supplementary Information

**Additional file 1: Figure S1.** Model building. The model classifies the total population N* into two sections. The first class is totally confined at their domicile and they are completely safe and cannot transmit the virus to the others. The second class is totally infected, free to move, and they are completely unsafe and can transmit the virus to the others. The social, economic, physical, sociological, psychiatric, etc. functions of the two classes are different. Due to the different functions, the model mathematically represents the total population as a complex number (N*). The real part is proportional to the number of confined individuals while the imaginary part represents the number of infected individuals. After the model, the confined class is passive, cannot transmit the virus via international channels; while the infected class is active, and can transmit the virus via internal and international channels. With the present model, the susceptible, the exposed, and the infectious cases are considered as infected cases; while the recovered cases are with the confined cases.

## Data Availability

All data are available in the main text and in the attached files with the method materials. All data, code, and materials used in the analysis are available in some form to any researcher for purposes of reproducing or/and extending the analysis.

## References

[1] Yang Y, Peng F, Wang R, Guan K, Jiang T, Guogang X, Sun J, Chang C (2020). The deadly coronaviruses: The 2003 SARS pandemic and the 2020 novel coronavirus epidemic in China. J Autoimmun.

[2] Guo Y-R, Cao Q-D, Hong Z-S, Tan Y-Y, Chen S-D, Jin H-J, Tan K-S, Wang D-Y, Yan Y (2020). The origin, transmission and clinical therapies on coronavirus disease 2019 (COVID-19) outbreak - an update on the status. Mil Med Res.

[3] COVID-19 Coronavirus Pandemic. Worldometer https://www.worldometers.info/coronavirus/ (2020).

[4] Coronavirus Disease 2019 (COVID-19) Situation Report 126 https://www.who.int/docs/default-source/coronaviruse/situation-reports/20200525-covid-19-sitrep-126.pdf?sfvrsn=887dbd66_2 (WHO, 2020).

[5] Lee V, Chiew C, Khong W (2020). Interrupting transmission of COVID-19: lessons from containment efforts in Singapore. J Travel Med.

[6] Anderson RM, Heesterbeek H, Klinkenberg D, Hollingsworth TD (2020). Comment, How will country-based mitigation measures influence the course of the COVID-19 epidemic?. Lancet.

[7] Janice HT, Erika H, Mark Z, Priyanka P, Paul S, Aser GR (2020). (2020) Covid-19: how doctors and healthcare systems are tackling coronavirus worldwide. BMJ.

[8] Seth F, Swapnil M, Axel G (2020). Estimating the number of infections and the impact of non-pharmaceutical interventions on COVID-19 in 11 European countries. Imperial College London.

[9] Rahman B, Aziz IA, Khdhr FW, Mahmood DFD (2020). Preliminary estimation of the basic reproduction number of SARS-CoV-2 in the Middle East. Bull World Health Organ.

[10] Adam JK, Timothy WR, Charlie D, Yang L, John E, Sebastian F, Rosalind ME (2020). Early dynamics of transmission and control of COVID-19: a mathematical modelling study. Lancet Infect Dis.

[11] Paul L, DelamaterErica J, StreetTimothy F, LeslieTimothy F, LeslieKathryn H, JacobsenKathryn H (2019). Jacobsen, Complexity of the Basic Reproduction Number (R0). Emerg Infect Dis.

[12] N. James MacLachlan, Antiviral Immunity and Prophylaxis, Chapter 4, Fenner's Veterinary Virology, 2011, pp. 275–291, 10.1016/B978-0-12-375158-4.00004-3, Fenner's Veterinary Virology, Fourth Edition, FENNER'S VETERINARY VIROLOGY, Fourth Edition, Edited by N. James MacLachlan

[13] Kenji M, Gerardo C (2020). Transmission potential of the novel coronavirus (COVID-19) onboard the diamond Princess Cruises Ship. Infect Dis Modelling.

[14] Peirlinck M, Linka K, Sahli Costabal F, Bendavid E, Bhattacharya J, Ioannidis JPA, Kuhl E (2020). Visualizing the invisible: The effect of asymptomatic transmission on the outbreak dynamics of COVID-19. medRxiv.

