# Supplementary material for: Successive waves of COVID 19: confinement effects on virus-prevalence with a mathematical model
Source: Eur J Med Res. 2021 Oct 30;26:128. doi: 10.1186/s40001-021-00596-6 (PMC8556837; doi:10.1186/s40001-021-00596-6)
Supplement: Supplementary file 1 — Additional file 1: Figure S1. Model building. The model classifies the total population N* into two sections. The first class is totally confined at their domicile and they are completely safe and cannot transmit the virus to the others. The second class is totally infected, free to move, and they are completely unsafe and can transmit the virus to the others. The social, economic, physical, sociological, psychiatric, etc. functions of the two classes are different. Due to the different functions, the model mathematically represents the total population as a complex number (N*). The real part is proportional to the number of confined individuals while the imaginary part represents the number of infected individuals. After the model, the confined class is passive, cannot transmit the virus via international channels; while the infected class is active, and can transmit the virus via internal and international channels. With the present model, the susceptible, the exposed, and the infectious cases are considered as infected cases; while the recovered cases are with the confined cases. [file 40001_2021_596_MOESM1_ESM.pdf]

Successive Waves of COVID 19: Confinement Effects on Virus-prevalence with a mathematical model

S. Abdalla*[1], D. Bakhshwin[2], W. Shirbeeny[1], Ahmad Bakhshwin[3], F.Bahabri[1, 4], Abdulaziz Bakhshwin[5], Samar M. Alsaggaf[6]

Materials and Methods

Epidemic buildup

For Covid-19, we will consider that the basic reproduction number in any population is greater than one. Therefore, the epidemic-buildup of Covid-19 (or any similar epidemic attack) rise exponentially [S1] from zero. For one wave attack, on writes

$$I(t) = I_\infty \left(1 - I_\infty e^{-\frac{t}{\tau}}\right) \tag{S1}$$

Here, $I(t)$ is the number of daily-infections at a certain time t, starting from zero-day ($t = 0$). I_∞ is the number of infected individuals when time tends to infinite values, with $I_\infty \neq 0$. We define τ as the time required the number of infected individuals to attain about third the total population N (I_τ = 36.789% N). At this condition, τ = t; equation (S1) becomes $I(t) = I_\infty(1 - I_\infty e^{-1})$, and the ratio $\frac{I(t)}{I_\infty} = (1 - e^{-1}) = 0.63212$. However, N = I_∞ + $I(t)$, therefore, at τ = t, $I(t)$ = 0.36789 N. This simple mathematical-note, means that any virus cannot extremist the human been. One believes that the natural immunity, in the absence of any vaccine or effective drug, plays the essential role to keep the human-race safe.

In addition, we call the time τ as epidemic relaxation time. τ is a function of different parameters as one will see later but it is not a function of time.

The limits of equation (1) are as following:

At zero time: $$I(0) = I_\infty \left(1 - I_\infty e^{-\frac{0}{\tau}}\right) = I_\infty(1 - I_\infty * 1) = 0 \tag{S2}$$

There is no infection at time zero

At infinite times: $$I(\infty) = I_\infty \left(1 - I_\infty e^{-\frac{0}{\tau}}\right) = I_\infty \left(1 - \frac{I_\infty}{\infty}\right) = I_\infty \tag{S3}$$

There is I_∞ infections at infinite times, which means that the model considers no complete decay of viruses. This means that the viruses will stay and individuals should found the most suitable scenario to cohabitate with it. The rate of buildup of an epidemic (rise of I daily infections) is obtained, by differentiating the previous equation as

$$\frac{dI(t)}{dt} = -I_\infty \left(-\frac{1}{\tau}\right) e^{-\frac{t}{\tau}} = \frac{I_\infty e^{-\frac{t}{\tau}}}{\tau} \tag{S4}$$

Substituting equation (1) in equation (2) and assuming that the total infection number is, only, due to the Covid-19, one gets

$$\frac{dI(t)}{dt} = \frac{I_\infty - I(t)}{\tau} \tag{S5}$$

Theoretically, the rate of infection increases with time until attaining all the population. However, due to the presence of mitigating (resisting) parameters (for example, vaccine, drugs,

lockdown or/and natural popular-immunity), this rate increases until reaching a maximum number I_{max}, where the virus spread out will equalized with the opposed mitigating conditions. Then *I* (t) starts to fall down, and finally, it reaches I_∞ at infinite times.

As on August 15, 2020, there are neither known vaccines nor effective drugs to stop the positive rate of the daily infections (rate) given by equation (S5). The decision-makers in a certain country have no choice, to stop COVID-19 except asking individuals to stay at home (confine). By this passive, but effective, protocol, the epidemic starts to go down in some countries.

However, it goes up in other countries depending on the rate of the human-confinement (blocking or social distancing) rate. One defines the number of confined individuals at initial time B_0, at infinite times B_∞ and at any time *t*, *B* (*t*). In addition, one assumes that *B* (*t*) varies exponentially with time in such a way to, partially, "oppose" *I* (*t*) with a certain phase angleφ. We write *B* (*t*) = C_B*log [N - *I* (*t*)]. The proportionality-constant, CB, is chosen to keep a similarity in the magnitude between *I* (*t*) and *B* (*t*) because *B* (*t*) is in the order of million individuals and I (t) about thousands. Both B (t) and I (t) differ in their phase.

Moreover, from the physical point of view, the function of confined individuals is by far different from the function of mobile (infected) individuals. We define this situation, in mathematical terms, as a complex number with the parameter ($i\omega$), where $i = \sqrt{-1}$ and omega is the following rate,

$$\omega = \frac{\partial}{\partial t}\left(\frac{B(t) - B_\infty}{I_\infty - I(t)}\right) \quad \text{(S6)}$$

Moreover, one describes the number of confined individuals at any time *B* (*t*) as a complex number in order to resist the rise of daily-infections given by equation (S1).

$$B^*(t) = B_\infty + (B_0 - B_\infty)e^{i\omega t} \quad \text{(S7)}$$

Equation (S1) and equation (S7), give

$$\tau\frac{dI(t)}{dt} = \quad (B_0 - B_\infty)e^{i\omega t} - I(t) \quad \text{(S8)}$$

The general solution of the first-order differential equation (S8) is

$$I(t) = Ce^{-\frac{t}{\tau}} + \frac{(B_0 - B_\infty)e^{i\omega t}}{1+i\omega t} \quad \text{(S9)}$$

Equation (S9) gives at infinite times (t → ∞), the maximum number of infected individuals$I(t) = Ce^{-\frac{\infty}{\tau}} + \frac{(B_0 - B_\infty)e^{i\omega\infty}}{1+i\omega\infty} = (B_0 - B_\infty)$ which leads to

$$I_\infty = (B_0 - B_\infty) \quad \text{(S10)}$$

At initial conditions (t = 0), $I_0 = Ce^{-\frac{0}{\tau}} + \frac{(B_0 - B_\infty)e^{i\omega*0}}{1+i\omega*0} = C + (B_0 - B_\infty)$

$$C = I_0 - (B_0 - B_\infty)$$

$$C = I_0 - I_\infty \quad \text{(S11)}$$

Equation (S9) becomes $$I(t) = (I_0 - I_\infty)e^{-\frac{t}{\tau}} + \frac{(B_0 - B_\infty)e^{i\omega t}}{1+i\omega t} \quad \text{(S12)}$$

At time *t*, sufficiently large when compared with τ, the first term on the right side of Equation (S12) becomes so small that it can be neglected, and we get the solution for *I* (t) as

$$I(t) = \frac{(B_0 - B_\infty)e^{i\omega t}}{1+i\omega t} \quad \text{(S13)}$$

$$I(t) = \left((B_\infty - 1)\left(\frac{B_0 - B_\infty}{1+i\omega t}\right)\right)e^{i\omega t} \quad \text{(S14)}$$

The number of total confined individuals should equal two parts: the infected individuals and the rest of the population, which are completely lockdown:

$$B^* e^{i\omega t} = (B_\infty - B_0)\, e^{i\omega t} + I(t) \quad \text{(S15)}$$

Equations 13S and 15S give

$$B(t) + i\omega t * I(t) = 1 + \left(B_\infty - 1 + \frac{B_0 - B_\infty}{1+i\omega t} \right) \quad \text{(S16)}$$

Equating the real and imaginary parts, we readily obtain

$$B(t) = B_\infty + \frac{B_0 - B_\infty}{1+(\omega t)^2} \quad \text{(S17)}$$

$$I(t) = \omega t \left(\frac{B_0 - B_\infty}{1+(\omega t)^2} \right) \quad \text{(S18)}$$

It is easy to show that

$$\tan\varphi = \omega\tau \left(\frac{B_0 - B_\infty}{B_0 + B_\infty(\omega t)^2} \right) \quad \text{(S19)}$$

Equations (S18) estimates the number of daily infections as a function of time starting from the initial point at *t* = zero. However, there is an important issue. The virus starts its first attack until the individuals detect its danger and start a mechanism of confinements. The period from the first infection and first blocking action takes delay interval of time δ (days). This important parameter affects drastically the future total infections, and even increases the probability to have another epidemic-attack. Therefore, we consider the time delay and modify equations (17S- 19S) in such a way to consider this important factor. Equations (17S- 19S) become,

$$B(t) = B_\infty + \frac{B_0 - B_\infty}{1+(\omega(t-\delta))^2} \quad \text{(S20)}$$

$$I(t) = \omega(t-\delta) \left(\frac{B_0 - B_\infty}{1+(\omega(t-\delta))^2} \right) \quad \text{(S21)}$$

$$\tan\varphi = \omega\tau \left(\frac{B_0 - B_\infty}{B_0 + B_\infty(\omega(t-\delta))^2} \right) \quad \text{(S22)}$$

In order to obtain the maximum number of infected individuals, one differentiate with respect to $\omega(t-\delta)$and then equalize to zero

$$\frac{\partial I(t)}{\partial t} = \frac{\partial}{\partial t}\left[(\omega t)\frac{B_0 - B_\infty}{1+(\omega(t-\delta))^2} - (\omega\delta)\left(\frac{B_0 - B_\infty}{1+(\omega(t-\delta))^2} \right) \right] \quad \text{(S23)}$$

$$\frac{\partial I(t)}{\partial t} = (B_0 - B_\infty)\left[\frac{(\omega(t-\delta))^2 - 1}{(1+\omega(t-\delta))^2} \right] = 0 \quad \text{(S24)}$$

Therefore, the maximum value of the curve given by equation (19) occurs either when $B_0 = B_\infty$ or at the condition$(\omega(t-\delta))^2 = 1$, which means that one gets a maximum when

$$t = \frac{1}{\omega} + \delta \quad \text{(S25)}$$

Equation (21) describes the number of daily infections for this wave. After the present model, there are different waves attack. The number of these waves, *m*, depend on δ and on the social-behaviour of individuals. The successive waves have an additive property; i.e. for *m* waves attack a certain country. The resultant of total waves,

$$B_{tot}(t) = \sum_{n=1}^{n=m} \left(B_{\infty n} + \frac{(B_0 - B_\infty)_n}{1+(\omega_n(t-\delta_n))^2} \right) \quad \text{(S26)}$$

$$I_{tot}(t) = \sum_{n=1}^{n=m} \omega_n(t-\delta_n) \left(\frac{(B_0 - B_\infty)_n}{1+(\omega_n(t-\delta_n))^2} \right) \quad \text{(S27)}$$

Equations (26) and (27) describe the number of confined and infected individuals attacked by *m* waves at duration interval *t*.

Possibility of second epidemic-attack after the first one:

We consider that the waves of first attack *I* (*t*) continue infecting individuals with a relaxation time τ_1. The second wave group have relaxation time τ_2. The rise of infection rate takes the two specific times τ_1 and τ_2 on the same time and it goes up in an exponential manner by the factor$e^{\frac{[\tau_2\tau_1]t}{[\tau_2+\tau_1]}}$.

$I_{\infty 2}$ is the limit number of infected individuals reported at infinite times.

$$I_2(t) = [I_1(t) - I_{\infty 2}]\left(1 - [I_1(t) - I_{\infty 2}]e^{-\frac{[\tau_2\tau_1]t}{[\tau_2+\tau_1]}}\right) \quad \text{(S28)}$$

The boundary conditions of equation (1): At zero time

$$I_2(0) = I_{02} = [I_{01}(t) - I_{\infty 2}](1 - [I_{01} - I_{\infty 2}] * 1) = 0 \quad \text{(S29)}$$

There is no infections, from the second-epidemic attack at time zero, of the second attack.
At infinite times:

$$I(\infty) = I_\infty\left(1 - I_\infty e^{-\frac{0}{\tau}}\right) = I_\infty\left(1 - \frac{I_\infty}{\infty}\right) = I_\infty \quad \text{(S30)}$$

There is $[iI_{\infty 2}]^2$ infections at infinite times. The complex number of the infinite times at second epidemic attack is due to presence of confined individuals from the first epidemic attack. In order to get the maximum number of infected individuals, one differentiates equation (S28) with respect to time

$$\frac{dI_2(t)}{dt} = \left(\frac{dI_1(t)}{dt}\right)\left(1 - [I_1(t) - I_{\infty 2}]e^{-\frac{[\tau_2\tau_1]t}{[\tau_2+\tau_1]}}\right) + [I_1(t) - I_{\infty 2}]\frac{d}{dt}\left(1 - [I_1(t) - I_{\infty 2}]e^{-\frac{[\tau_2\tau_1]t}{[\tau_2+\tau_1]}}\right) \text{(31)}$$

Substituting equation (S28) in equation (S31), one gets

$$\frac{dI_2(t)}{dt} = \left(\frac{I_{\infty 1}e^{-\frac{t}{\tau_1}}}{\tau_1}\right)\left(1 - [I_1(t) - I_{\infty 2}]e^{-\frac{[\tau_2\tau_1]t}{[\tau_2+\tau_1]}}\right) + [I_1(t) - I_{\infty 2}]\left[\frac{I_{\infty 1}e^{-\frac{t}{\tau_1}}}{\tau_1}e^{-\frac{[\tau_2\tau_1]t}{[\tau_2+\tau_1]}} + [I_{\infty 1}\left(1 - I_{\infty 1}e^{-\frac{t}{\tau_1}}\right) - I_{\infty 2}]\frac{[\tau_2\tau_1]}{[\tau_2+\tau_1]}e^{-\frac{[\tau_2\tau_1]t}{[\tau_2+\tau_1]}}\right] \quad \text{(S32)}$$

One defines the number of confined individuals at initial time B_{02}, at infinite times $B_{\infty 2}$ and at any time t, B_2 (t). In addition, one assumes that B_2 (t) varies exponentially with time in such a way to impede I_2 (t) with a certain phase angleφ_2. Moreover, from the physical point of view, the function of confined individuals by far different from the function of mobile (infected) individuals. From a mathematical point of view, this is written as a complex number with the parameter ($i\omega$), where $i = \sqrt{-1}$ and $\omega\tau_2$ the time necessary that the second epidemic-attack reaches 0.36789 of its maximum infected number. ω_2 is the frequency of second epidemic-attack; it is proportional to the relaxation time τ_2 of the wave.

$$\omega_1 = \frac{\partial}{\partial t}\left(\frac{B_1(t) - B_{\infty 1}}{I_{\infty 1} - I_1(t)}\right) \text{ and} \omega_2 = \frac{\partial}{\partial t}\left(\frac{B_2(t) - B_{\infty 2}}{I_{\infty 2} - I_2(t)}\right) \quad \text{(S33)}$$

For the second epidemic-attack, one describes the number of confined individuals at any time $B_2(t)$ as complex number. The real part represents the confined individuals. These individuals resist the rise of infections given by equation (28) as

$$B_2{}^*(t) = B_{\infty 2} + (B_{02} - B_{\infty 2})e^{\frac{[\tau_2\tau_1]t}{[\tau_2+\tau_1]}} \quad \text{(S34)}$$

This model assumes that the second epidemic-attack attacks by successive waves with epidemic relaxation time τ_2. The leakage of confined individuals (going out from lockdown) means turning out to be infected. Thus, equation (28) and equation (32), give

$$\frac{\tau_1\tau_2\frac{dI_1(t)}{dt}\frac{dI_2(t)}{dt}}{\tau_1\frac{dI_1(t)}{dt} + \tau_2\frac{dI_2(t)}{dt}} = (B_0 - B_\infty)e^{i\omega t} - I_1(t) - I_2(t) \quad \text{(S33)}$$

The general solution of the last differential equation is

$$I_2(t) = \frac{C_1 e^{-\frac{t}{\tau_1}} C_1 e^{-\frac{t}{\tau_2}} + \frac{(B_{01} - B_{\infty 1})e^{i\omega_1 t}}{1+i\omega_2 t}\frac{(B_{02} - B_{\infty 2})e^{i\omega_2 t}}{1+i\omega_2 t}}{C_1 e^{-\frac{t}{\tau_1}} + C_1 e^{-\frac{t}{\tau_2}} + \frac{(B_{01} - B_{\infty 1})e^{i\omega_1 t}}{1+i\omega_1 t} + \frac{(B_{02} - B_{\infty 2})e^{i\omega_2 t}}{1+i\omega_2 t}} \quad \text{(S34)}$$

Taking into consideration the boundary conditions given at zero and infinite times by equations (2) and (3), one gets C_1 = C_2 = zero. Therefore, the solution of equation (33) is as following

$$I_2(t) = \frac{\frac{(B_{01} - B_{\infty 1})e^{i\omega_1 t}}{1+i\omega_2 t}\frac{(B_{02} - B_{\infty 2})e^{i\omega_2 t}}{1+i\omega_2 t}}{\frac{(B_{01} - B_{\infty 1})e^{i\omega_1 t}}{1+i\omega_1 t}+\frac{(B_{02} - B_{\infty 2})e^{i\omega_2 t}}{1+i\omega_2 t}} \quad \text{(S35)}$$

At the second epidemic-attack, the number total confined individuals should equal two parts the leaked individuals (infected) and the rest of population, which are completely lockdown:

$$B^* \frac{e^{i\omega_1 t}e^{i\omega_2 t}}{e^{i\omega_1 t} + e^{i\omega_2 t}} = (B_\infty - B_0)\frac{e^{i\omega_1 t}e^{i\omega_2 t}}{e^{i\omega_1 t} + e^{i\omega_2 t}} + I_1(t) + I_2(t) \quad \text{(S36)}$$

Equations 35 and 36 give

$$B^*(t) + i\omega t * [I_1(t) + I_2(t)] = 1 + \left(B_{\infty 2} - 1 + \frac{\frac{B_{01} - B_{\infty 1}}{1+i\omega_1 t} * \frac{B_{02} - B_{\infty 2}}{1+i\omega_2 t}}{\frac{B_{01} - B_{\infty 1}}{1+i\omega_1 t} + \frac{B_{02} - B_{\infty 2}}{1+i\omega_2 t}} \right) \quad \text{(S37)}$$

Equating the real and imaginary parts, we readily obtain

$$B_2(t) = B_{\infty 2} + \frac{\frac{B_{01} - B_{\infty 1}}{1+(\omega_1 t)^2} * \frac{B_{02} - B_{\infty 2}}{1+(\omega_2 t)^2}}{\frac{B_{01} - B_{\infty 1}}{1+(\omega_1 t)^2} + \frac{B_{02} - B_{\infty 2}}{1+(\omega_2 t)^2}} \quad \text{(S38)}$$

$$I_2(t) = \frac{(\omega_1 t)(\omega_2 t)}{(\omega_1 t) + (\omega_2 t)} \frac{\frac{B_{01} - B_{\infty 1}}{1+(\omega_1 t)^2} * \frac{B_{02} - B_{\infty 2}}{1+(\omega_2 t)^2}}{\frac{B_{01} - B_{\infty 1}}{1+(\omega_1 t)^2} + \frac{B_{02} - B_{\infty 2}}{1+(\omega_2 t)^2}} \quad \text{(S39)}$$

It is easy to show that

$$\tan\varphi = \frac{(\omega_1 t)(\omega_2 t)}{(\omega_1 t)+(\omega_2 t)} \left(\frac{B_{01} - B_{\infty 2}}{B_{01} + B_{\infty 2}\left(\frac{(\omega_1 t)(\omega_2 t)}{(\omega_1 t)+(\omega_2 t)}\right)^2} \right) \quad \text{(S40)}$$

Equations 38 and 39 -16 give the number of confined and infected individuals as a function of time starting from initial point at $t = 0$. However, the virus starts its attack until the responsible(s) detect its danger and arrange to start a mechanism of blockage. The period from first infection and first blocking action takes an interval of time Δ (days). This important parameter affects drastically the future total infections. One take this important factor into equations (38, 39)

$$B_2(t) = B_{\infty 2} + \left\{ \frac{\frac{B_{01} - B_{\infty 1}}{1+(\omega_1 (t-\Delta_1))^2} * \frac{B_{02} - B_{\infty 2}}{1+(\omega_2 (t-\Delta_2))^2}}{\left(\frac{B_{01} - B_{\infty 1}}{1+(\omega_1 (t-\Delta_1))^2}\right) + \left(\frac{B_{02} - B_{\infty 2}}{1+(\omega_2 (t-\Delta_2))^2}\right)} \right\} \quad \text{(S41)}$$

$$I_2(t) = \frac{(\omega_1 t)(\omega_2 t)}{(\omega_1 t) + (\omega_2 t)} \frac{\frac{B_{01} - B_{\infty 1}}{1+(\omega_1 (t-\Delta_1))^2} * \frac{B_{02} - B_{\infty 2}}{(\omega_2 (t-\Delta_2))^2}}{\frac{B_{01} - B_{\infty 1}}{1+(\omega_1 (t-\Delta_1))^2} + \frac{B_{02} - B_{\infty 2}}{(\omega_2 (t-\Delta_2))^2}} \quad \text{(S42)}$$

Equation (S41) describes the number of confined individuals for a certain wave and Equation (S42) describes the number of infected individuals for this wave. After the present model, the waves have additive property. For n waves attack certain country, for total waves, one writes:

$$B_2(t) = \sum_{n=1}^{z} B_{\infty 2n} + \left\{ \frac{\frac{B_{01n} - B_{\infty 1n}}{1 + (\omega_{1n}(t - \Delta_{1n}))^2} * \frac{B_{02n} - B_{\infty 2n}}{1 + (\omega_{2n}(t - \Delta_{2n}))^2}}{\left(\frac{B_{01n} - B_{\infty 1n}}{1 + (\omega_{1n}(t - \Delta_{1n}))^2}\right) + \left(\frac{B_{02n} - B_{\infty 2n}}{1 + (\omega_{2n}(t - \Delta_{2n}))^2}\right)} \right\} \tag{S43}$$

$$I_2(t) = \sum_{n=1}^{z} \frac{(\omega_1 t)(\omega_2 t)}{(\omega_1 t) + (\omega_2 t)} \left\{ \frac{\frac{B_{01n} - B_{\infty 1n}}{1 + (\omega_{1n}(t - \Delta_{1n}))^2} * \frac{B_{02n} - B_{\infty 2n}}{1 + (\omega_{2n}(t - \Delta_{2n}))^2}}{\left(\frac{B_{01n} - B_{\infty 1n}}{1 + (\omega_{1n}(t - \Delta_{1n}))^2}\right) + \left(\frac{B_{02n} - B_{\infty 2n}}{1 + (\omega_{2n}(t - \Delta_{2n}))^2}\right)} \right\} \tag{S44}$$

Equations (S44) estimates the number of daily infections attacked by z the waves at time t.

References and notes of supplementary materials

[S1] Joris Lammers, Jan Crusius, and Anne Gast, Correcting misperceptions of exponential coronavirus growth increases support for social distancing, PNAS July 14, 2020 117 (28) 16264-16266; first published June 24, 2020 https://doi.org/10.1073/pnas.2006048117

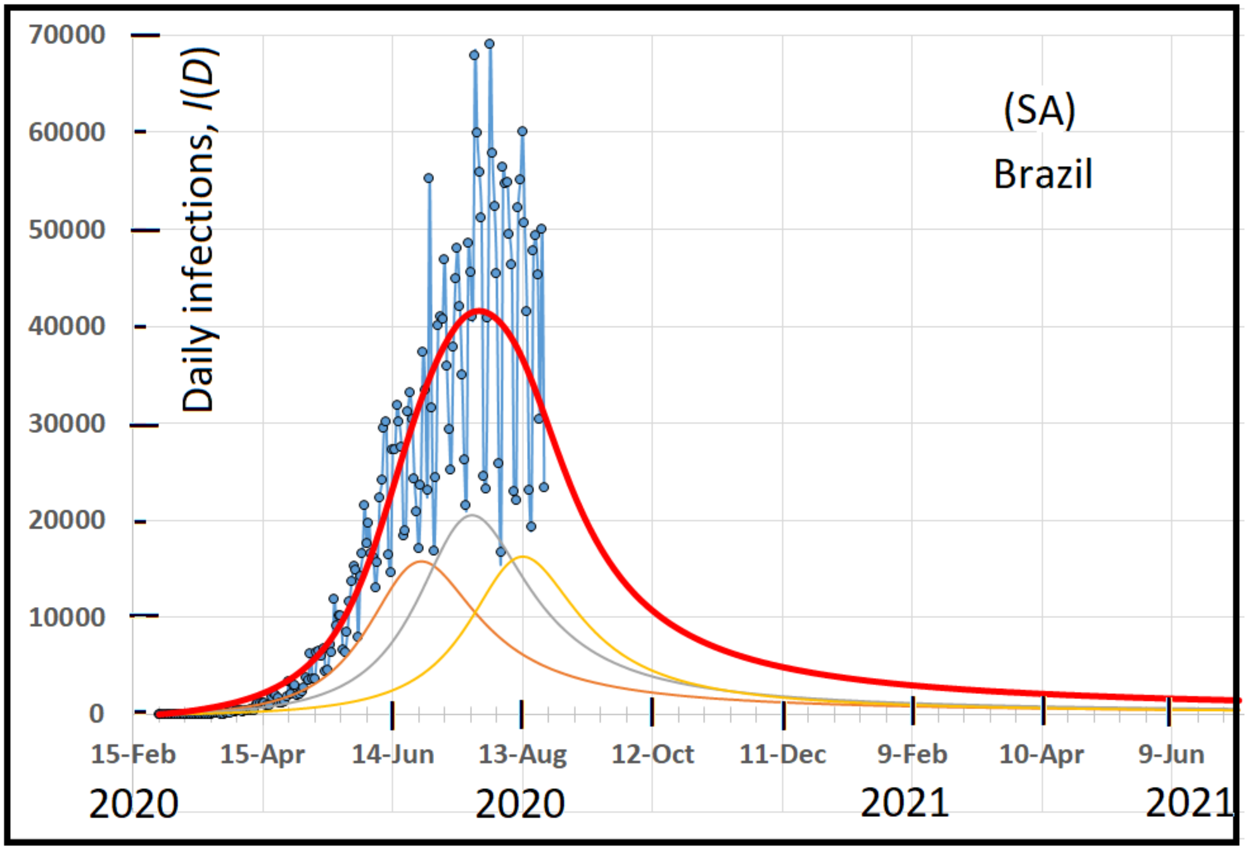

Fig. S1. (SA).

The daily infection of COVID 19 *I* (t) as a function of time, in Brazil. Blue circles are published data and continuous thick-red line represent the resultant of three successive waves shown as fine coulored lines and calculated after equation (4). One notes the similarity of the three waves around the wave summit.

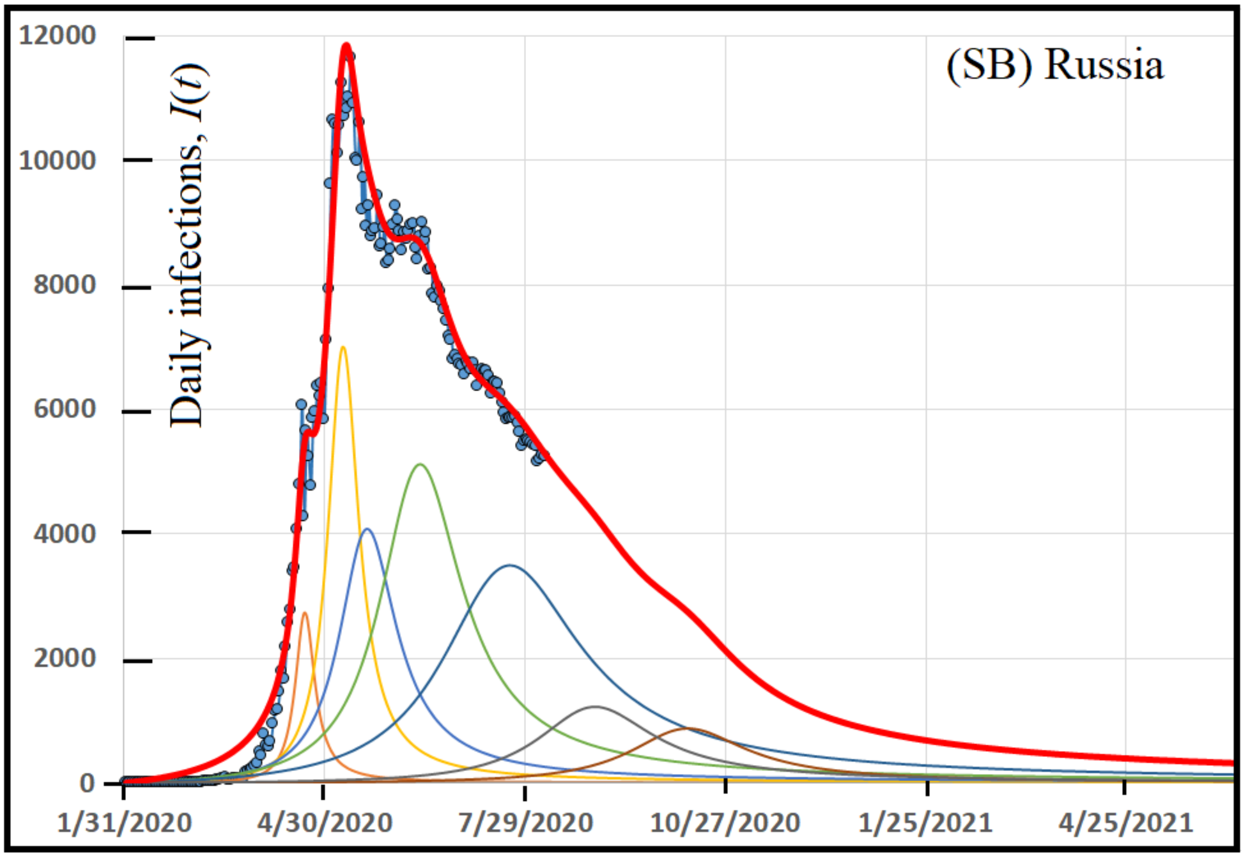

Fig. S2. (SB)

The daily infection of COVID 19 *I* (t) as a function of time, in Russia. Blue circles are published data and continuous thick-red line represent the resultant of seven successive waves shown as fine coulored lines and calculated after equation (4). We observe nearly linear decline of infections with time after the summit wave, in particular after June 14, 2020.

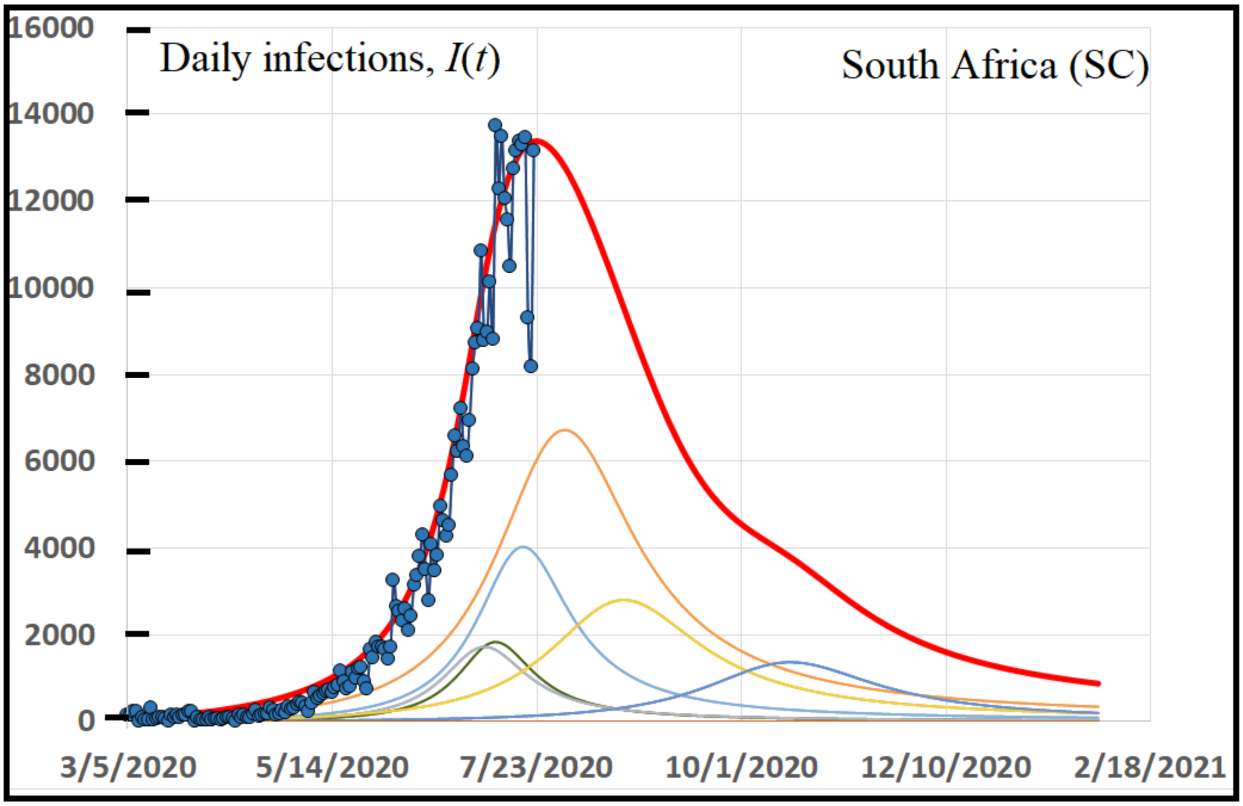

Fig. S3. (SC)

The daily infection of COVID 19 *I* (t) as a function of time, in South Africa. Blue circles are published data and continuous thick-red line represent the resultant of six successive waves shown as fine coulored lines and calculated after equation (4). The fitting processes show three waves before the summit and three others after the summit. After the present model, the area under wave-curves should be equal around the wave summit.

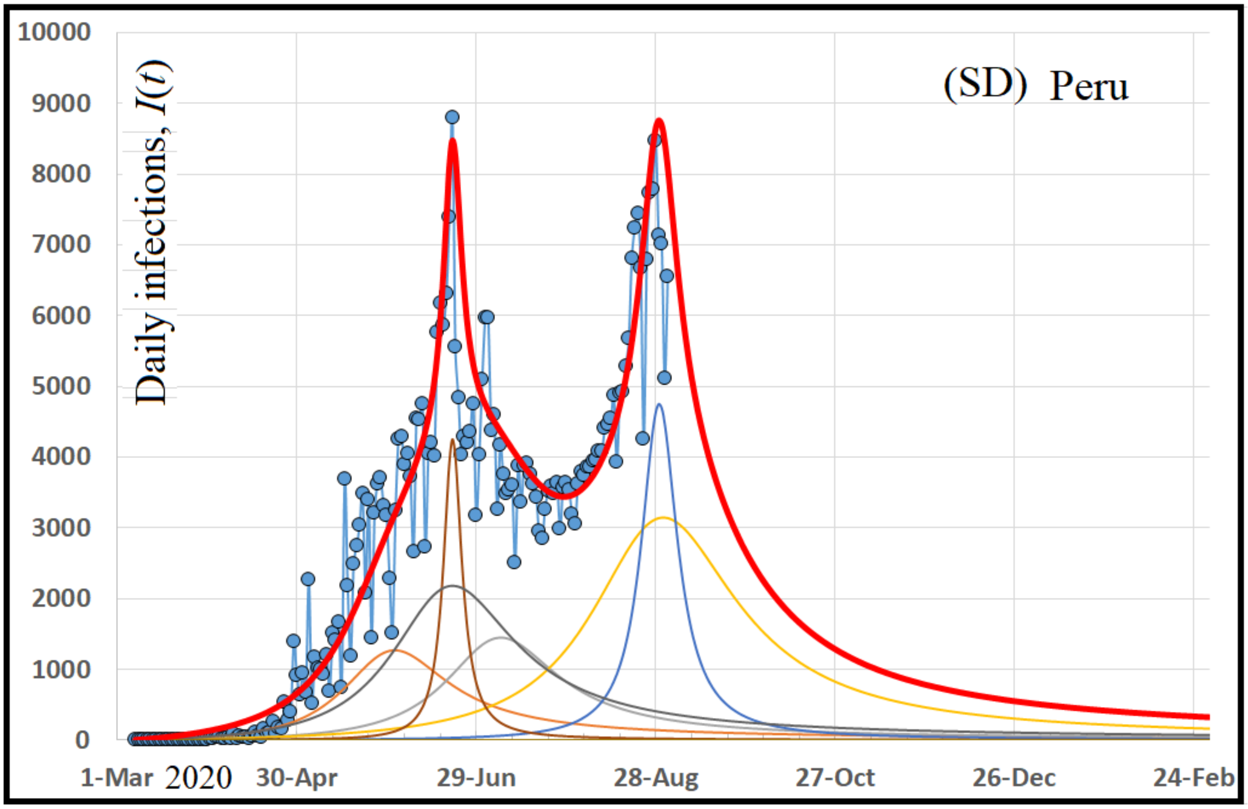

Fig. S4. (SD)

The daily infection of COVID 19 I (t) as a function of time, in Peru. Blue circles are published data and continuous thick-red line represent the resultant of six successive waves shown as fine coulored lines and calculated after equation (4). One notices the presence of two epidemic attacks; the onset one happened on June 21, 2020 and the second hit on August 26, 2020. The reason of triggering the second is unknown such as the onset one. However, when the social distancing is ignored, the virus would trigger the second hit as shown, in Peru, in August 26.

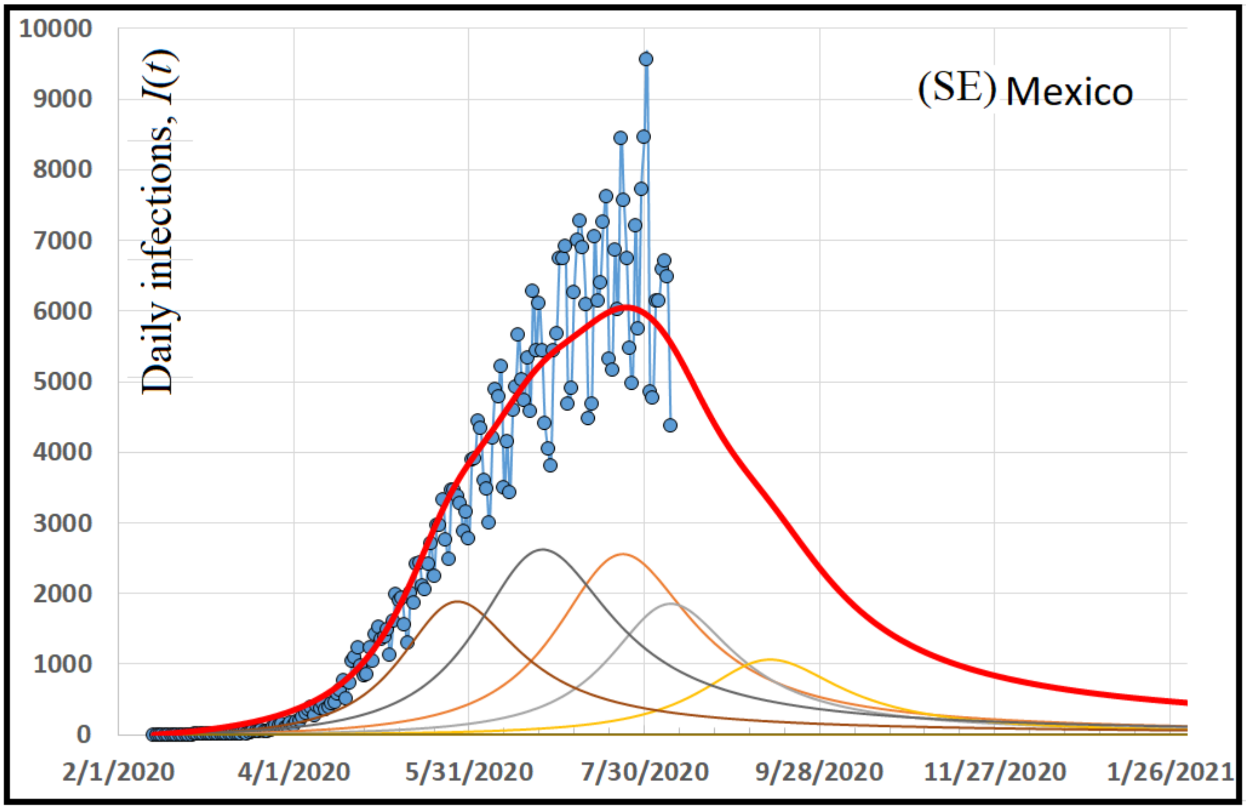

Fig. S5. (SE)

The daily infections of COVID 19 *I* (t) as a function of time, in Mexico. Blue circles are published data and continuous thick-red line represent the resultant of five successive waves shown as fine coulored lines and calculated after equation (4). The wave similarity around the summit of the resultant wave (red wave) is verified in the equality of the wave-areas around the red wave.

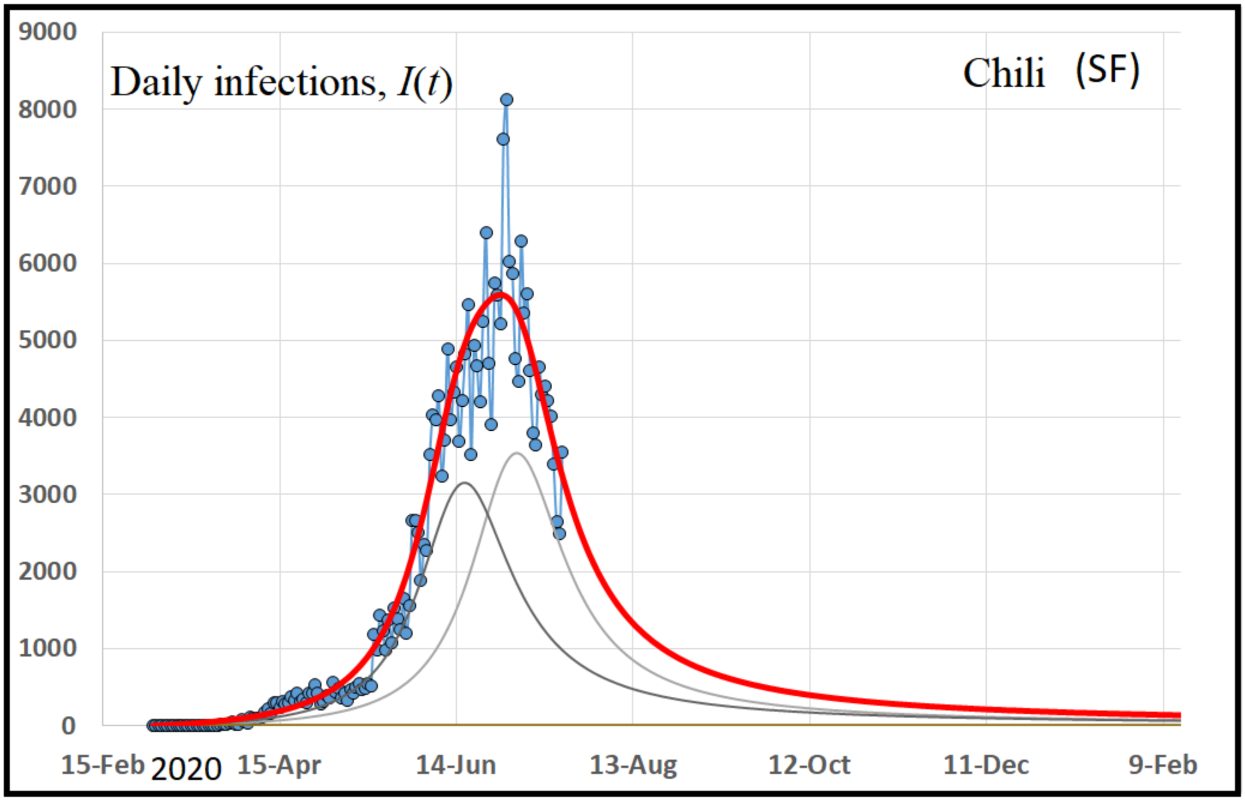

Fig. S6. (SF)

The daily infections of COVID 19 *I* (t) as a function of time, in Chile. Blue circles are published data and continuous thick-red line represent the resultant of two successive waves shown as fine coulored lines and calculated after equation (4).

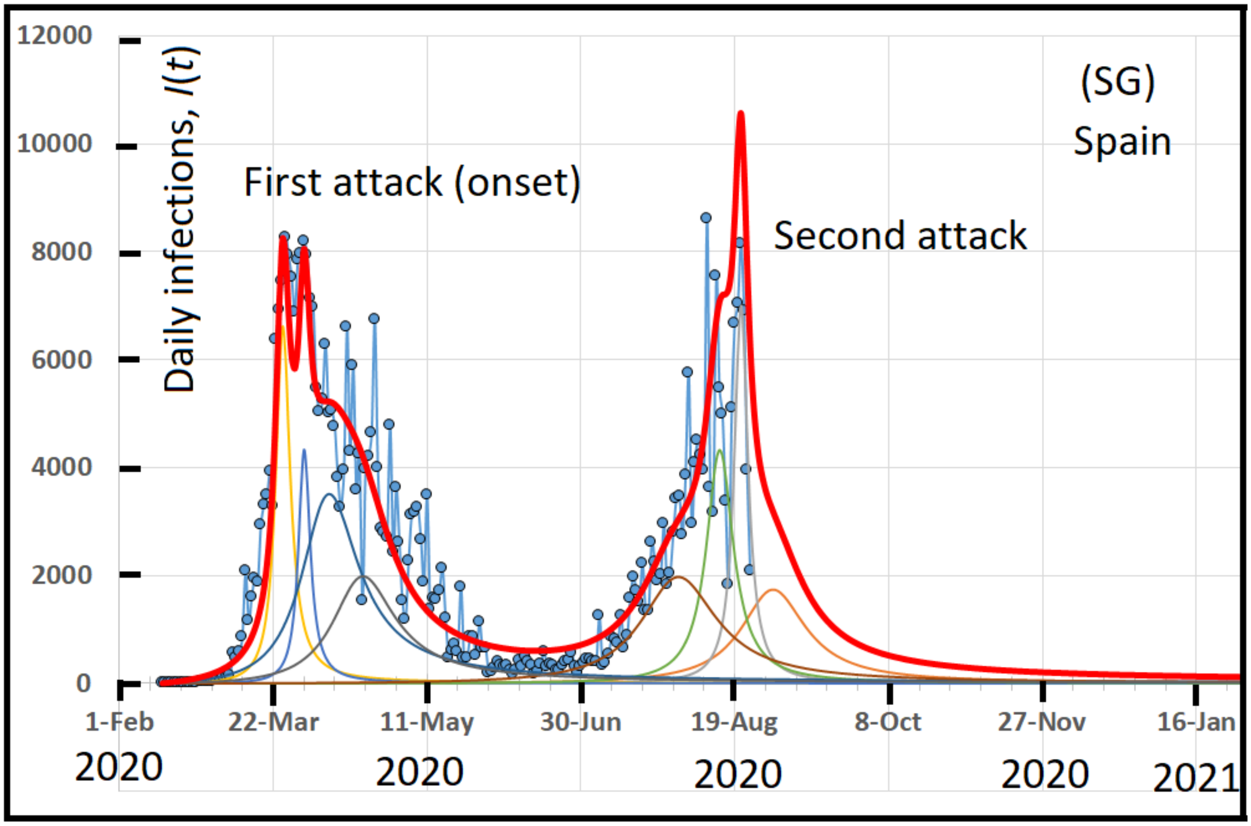

Fig. S7. (SG)

The daily infections of COVID 19 I (t) as a function of time, in Spain. Blue circles are published data and continuous thick-red line represent the resultant of three successive waves shown as fine coulored lines and calculated after equation (4). The onset attack has a calculated maximum of 8235 infections on March 11, 2020; the second attack happened with maximum infections of 10562, on August 19, 2020.

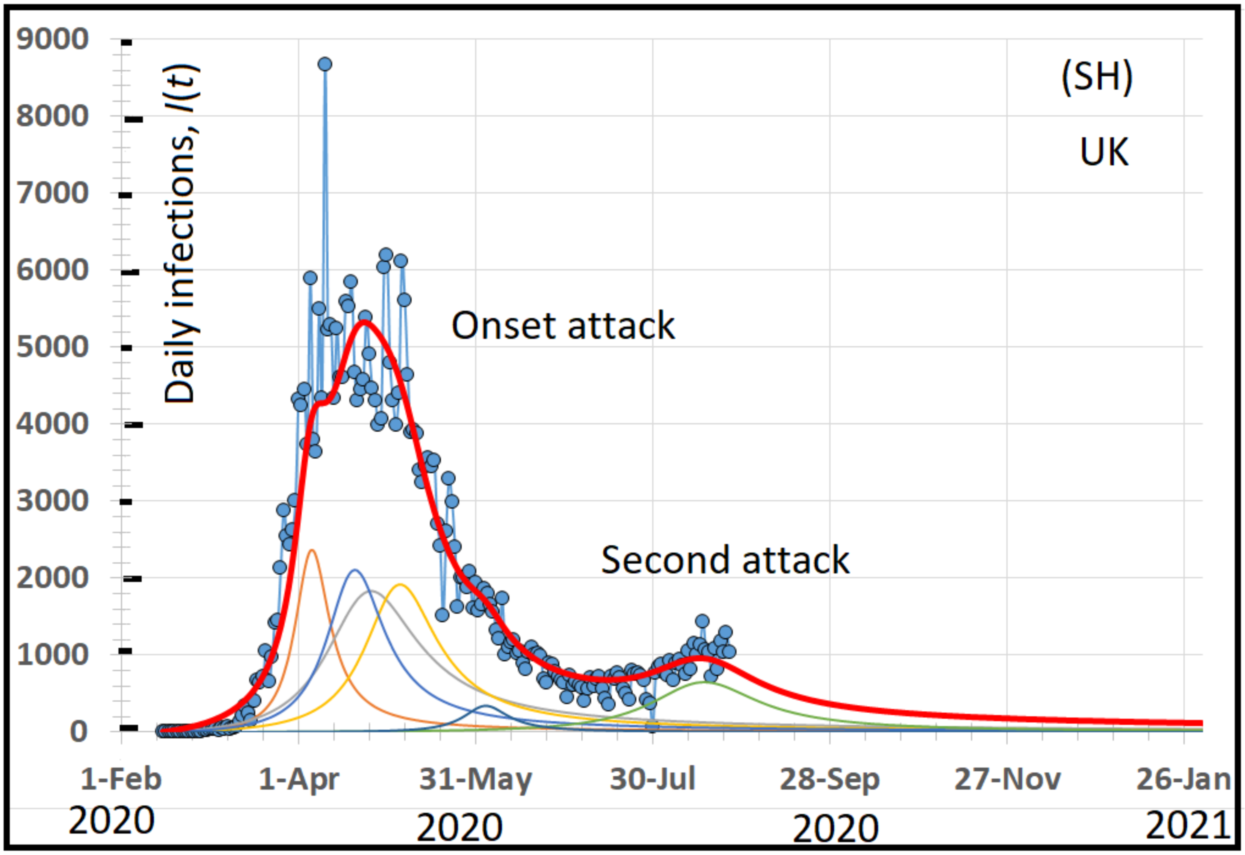

Fig. S8. (SH)

The daily infections of COVID 19 *I* (t) as a function of time, in United Kingdom. Blue circles are data and continuous thick-red line represent the resultant of five successive waves shown as fine coulored lines and calculated after equation (4). The onset attack has a calculated maximum of 5326 infections on April 23, 2020; the second attack has happened of 956 infections on August 17, 2020.

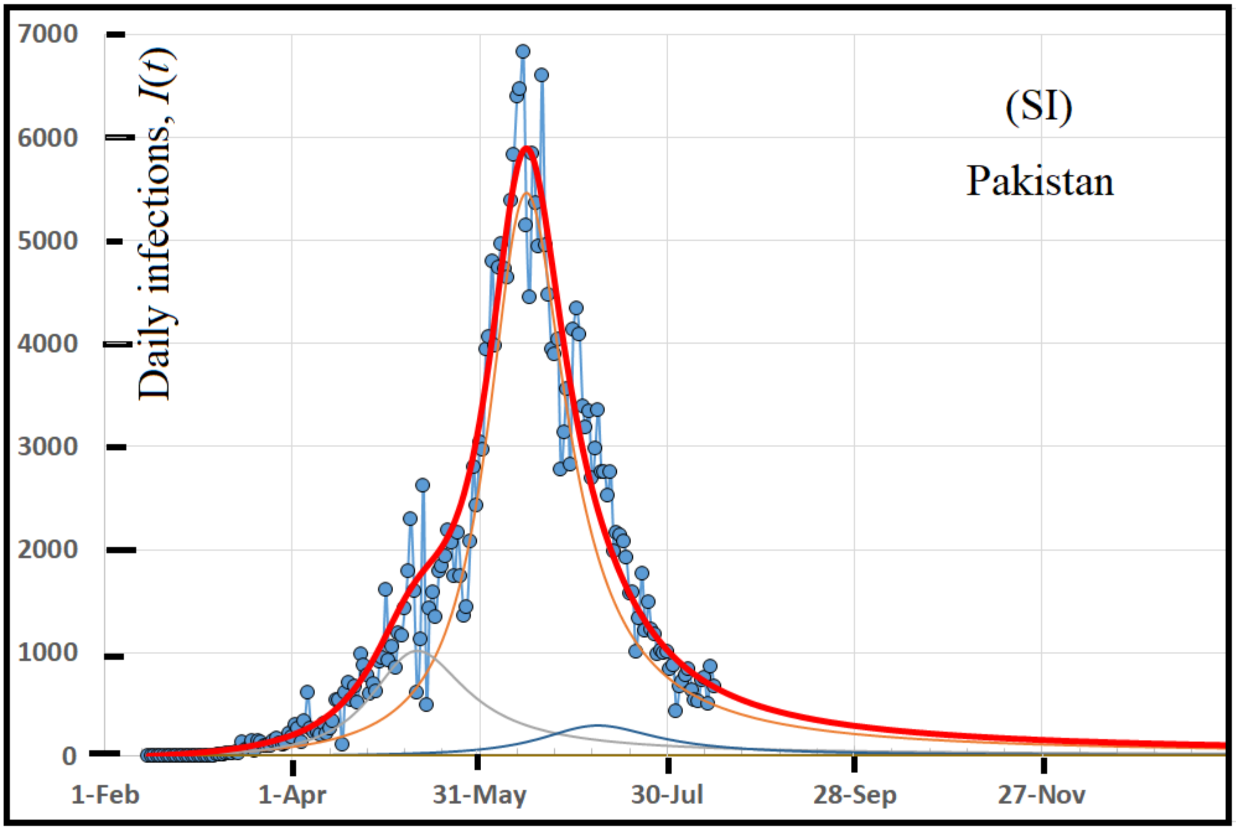

Fig. S9. (SI)

The daily infections of COVID 19 I (t) as a function of time, in Pakistan. Blue circles are published data and continuous thick-red line represent the resultant of three successive waves shown as fine coulored lines and calculated after equation (4).

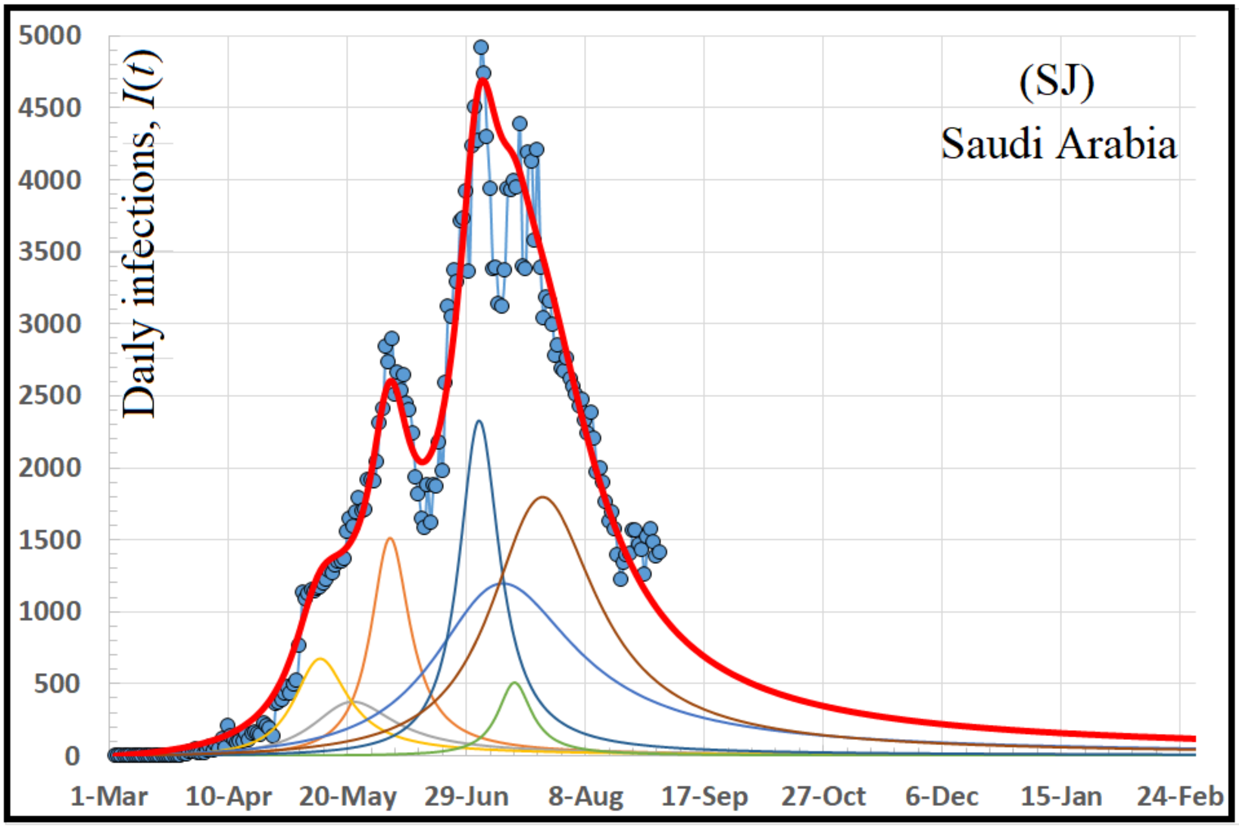

Fig. S10. (SJ)

The daily infections of COVID 19 *I* (t) as a function of time, in Saudi Arabia. Blue circles are published data and continuous thick-red line represent the resultant of seven successive waves shown as fine coulored lines and calculated after equation (4). The onset attack has a calculated maximum of 2502 infections on June 6, 2020; the second attack has happened of 4641 infections on July 27, 2020.

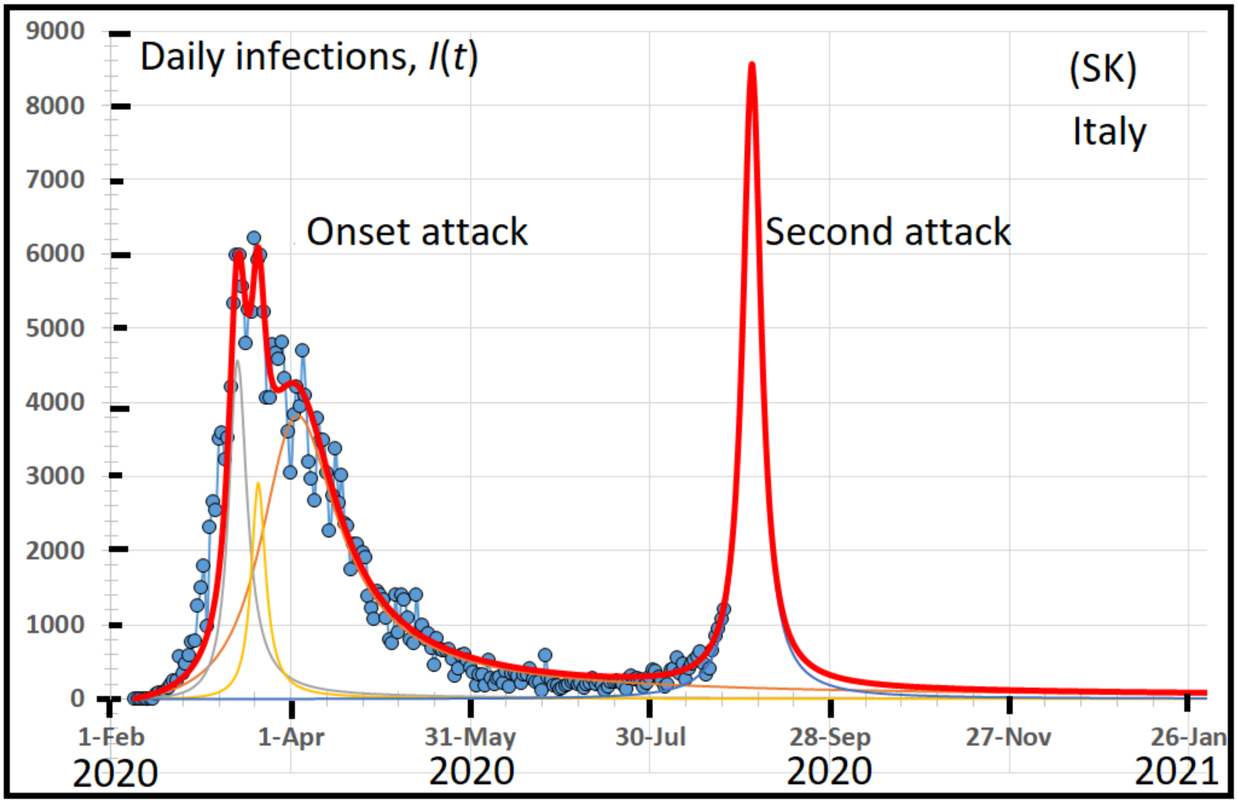

Fig. S11. (SK)

The daily infections of COVID 19 *I* (t) as a function of time, in Italy. Blue circles are published data and continuous thick-red line represent the resultant of three successive waves shown as fine coulored lines and calculated after equation (4). The onset attack has a calculated maximum of 6024 infections on March 21, 2020; the second attack will happened of 8441 infections on August 30, 2020.

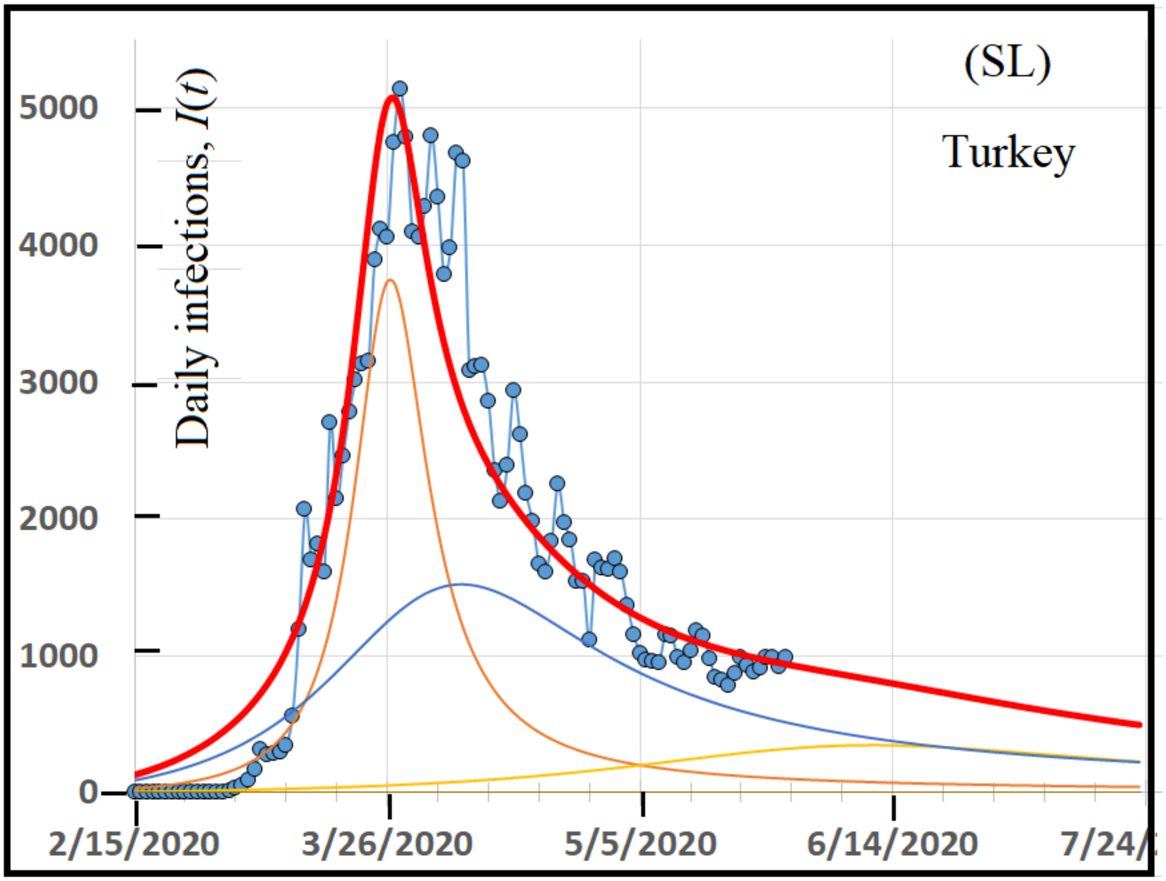

Fig. S12. (SL)

The daily infections of COVID 19 *I* (t) as a function of time, in Turkey. Blue circles are published data and continuous thick-red line represent the resultant of three successive waves shown as fine coulored lines and calculated after equation (4).

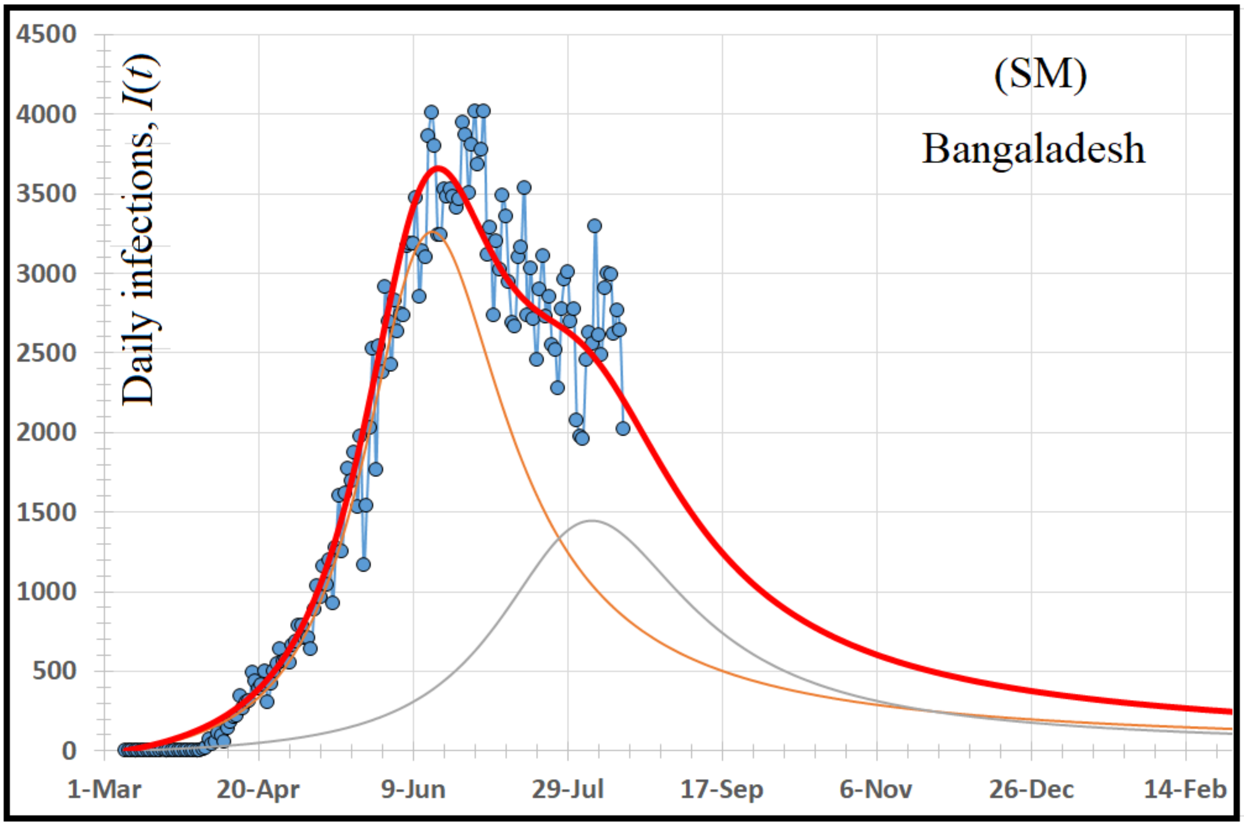

Fig. S13. (SM)

The daily infections of COVID 19 *I* (t) as a function of time, in Bangladesh. Blue circles are published data and continuous thick-red line represent the resultant of two wave shown as fine coulored lines and calculated after equation (4).

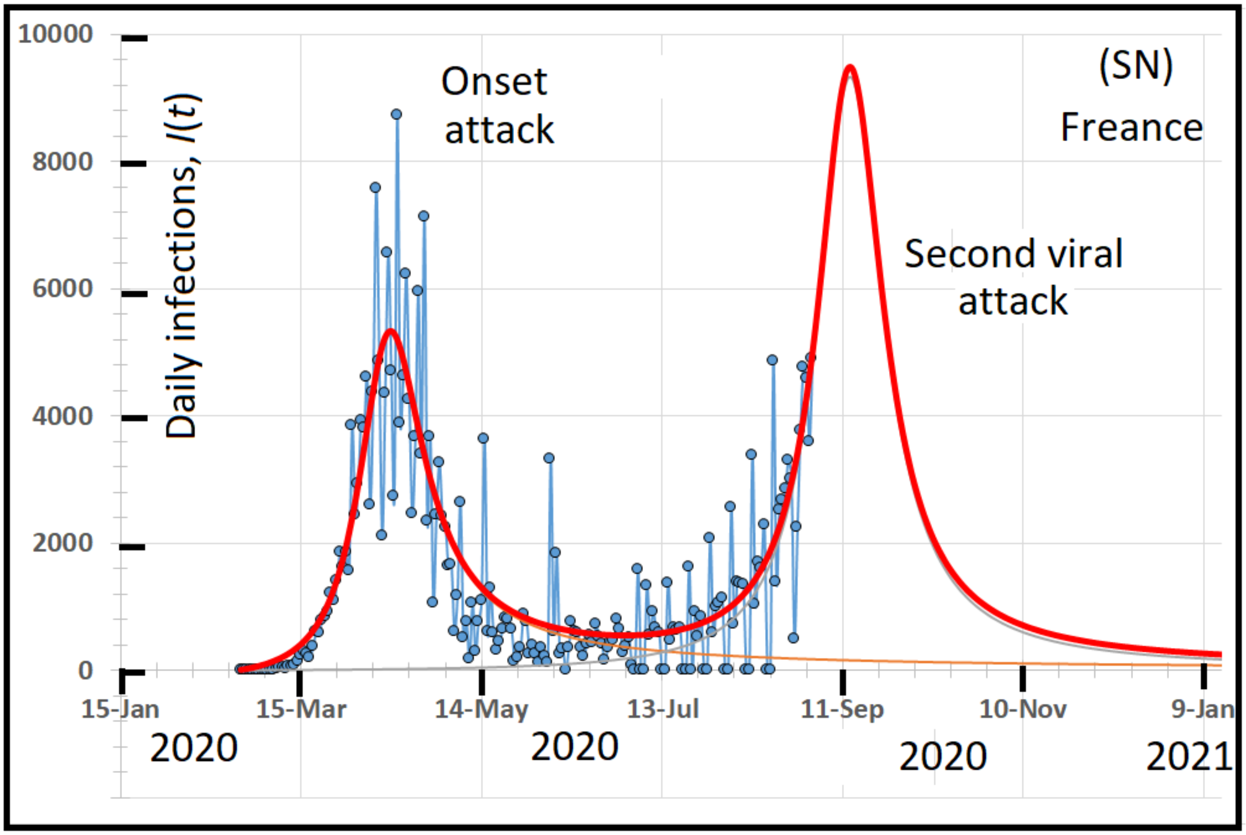

Fig. S14. (SN)

The daily infections of COVID 19 *I* (t) as a function of time, in France. Blue circles are published data and continuous red line with yellow circles represent the resultant of three successive waves shown as fine coulored lines and calculated after equation (4). The onset attack has a calculated maximum of 5292 infections on April 15, 2020; the second attack will happened of 9475 infections on September 14, 2020.

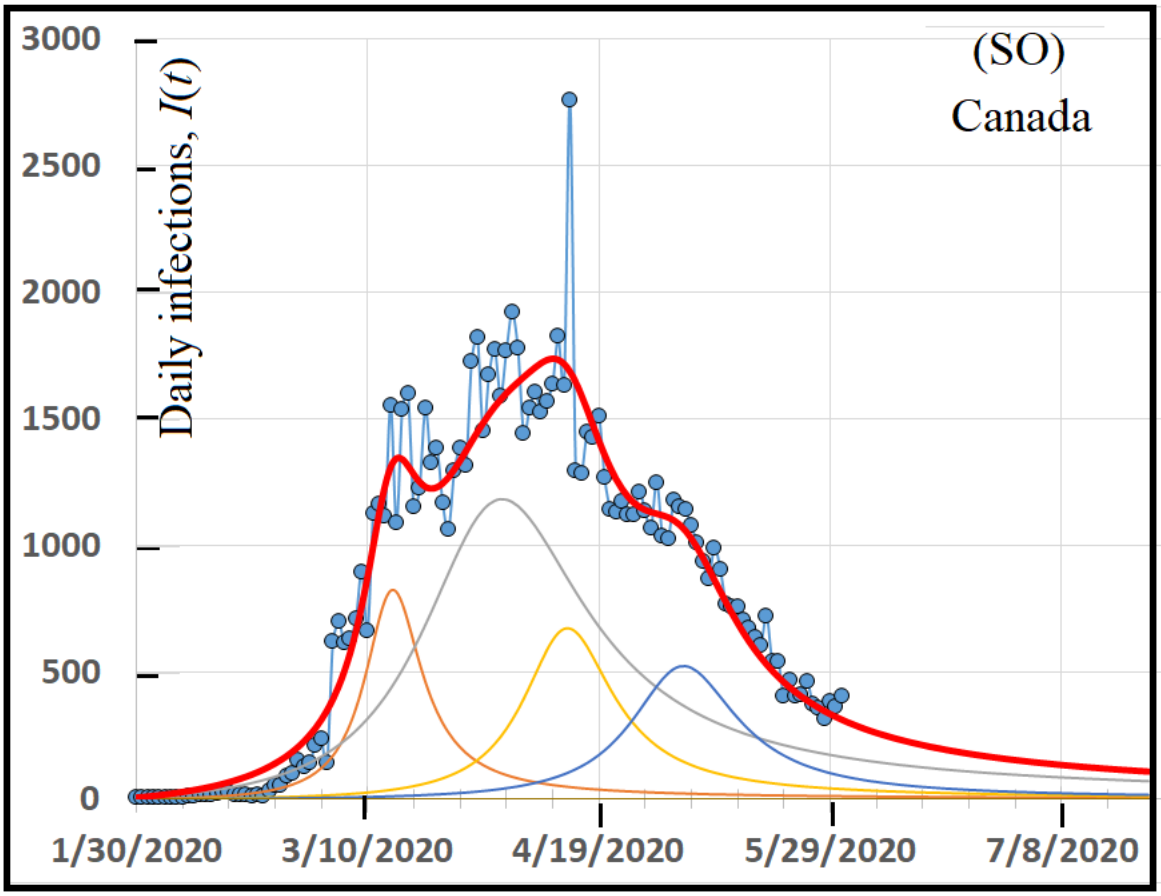

Fig. S15. (SO)

The daily infections of COVID 19 *I* (t) as a function of time, in Canada. Blue circles are published data and continuous red line with yellow circles represent the resultant of four successive waves shown as fine coulored lines and calculated after equation (4).

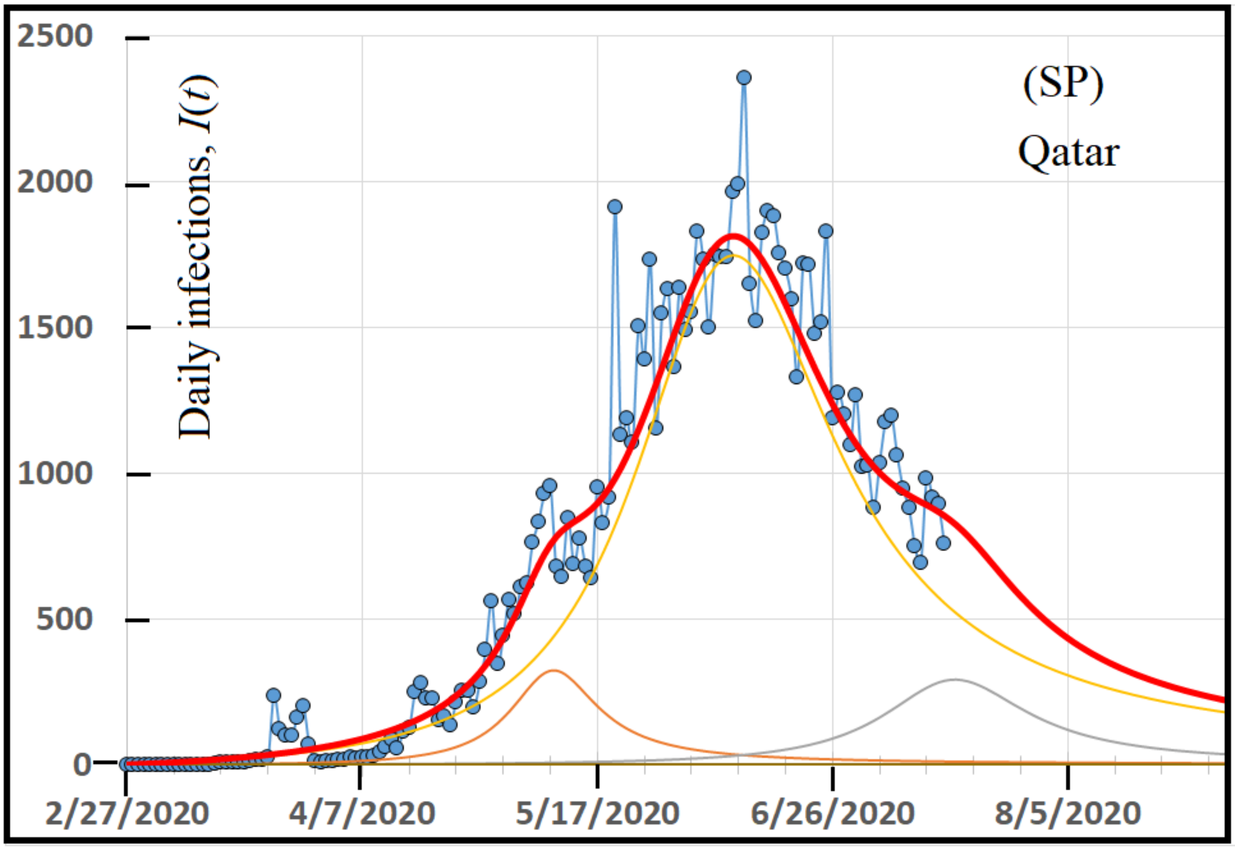

Fig. S16.

The daily infections of COVID 19 *I* (t) as a function of time, in Qatar. Blue circles are published data and continuous red line represents the resultant of three successive waves shown as fine coulored lines and calculated after equation (4). One can notice the area equality around the wave summit.

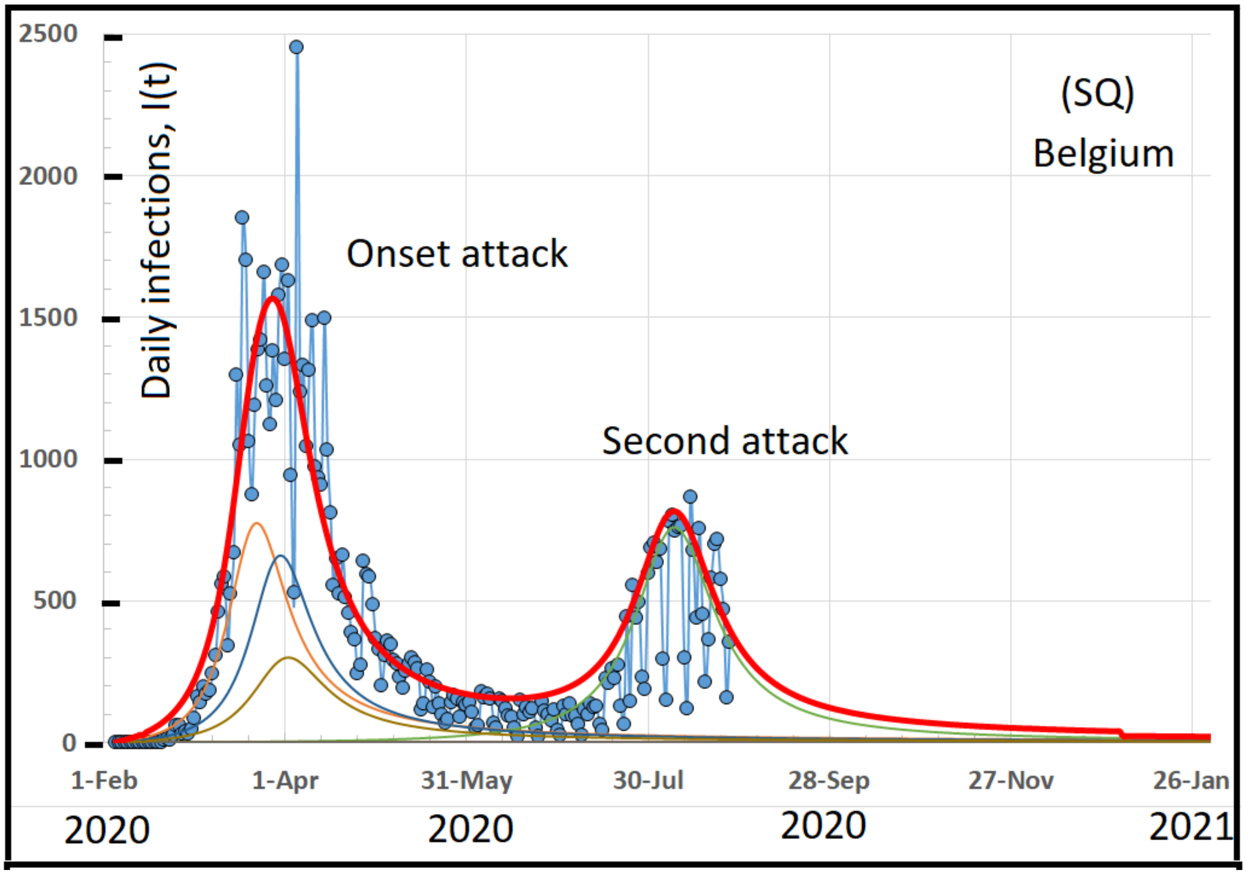

Fig. S17.

The daily infections of COVID 19 *I* (t) as a function of time, in Belgium. Blue circles are published data and continuous red line represents the resultant of three successive waves shown as fine coulored lines and calculated after equation (4).). The onset attack has a calculated maximum of 1567 infections on March 28, 2020; the second attack has happened of 815 infections on August 8, 2020.

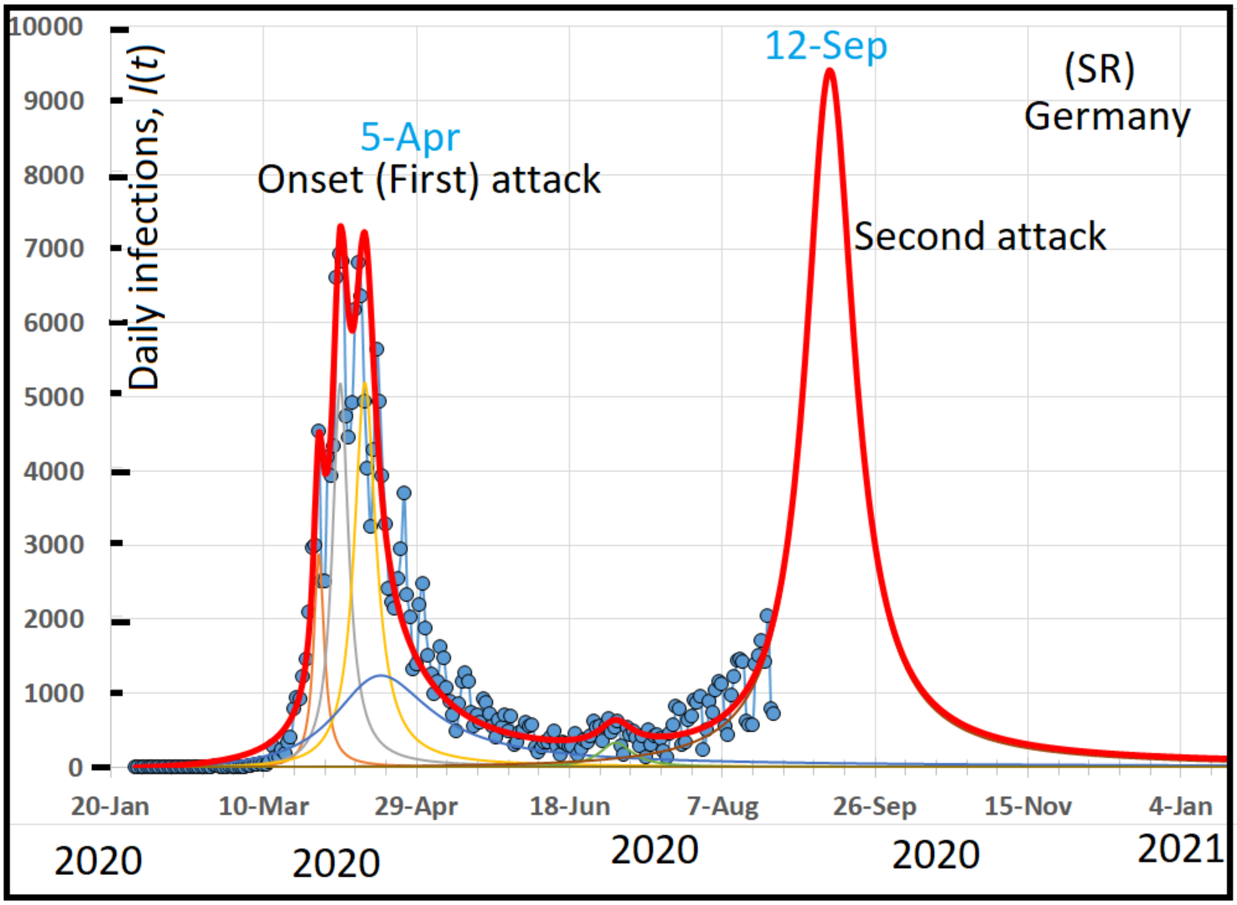

Fig. S18.

The daily infection of COVID 19 *I* (t) as a function of time, in Germany. Blue circles are published data and continuous red line represents the resultant of seven successive waves shown as fine coulored lines and calculated after equation (4). The onset attack has a calculated maximum of 7297 infections on April 4, 2020; the second attack will happened of 9415 infections on September 11, 2020.

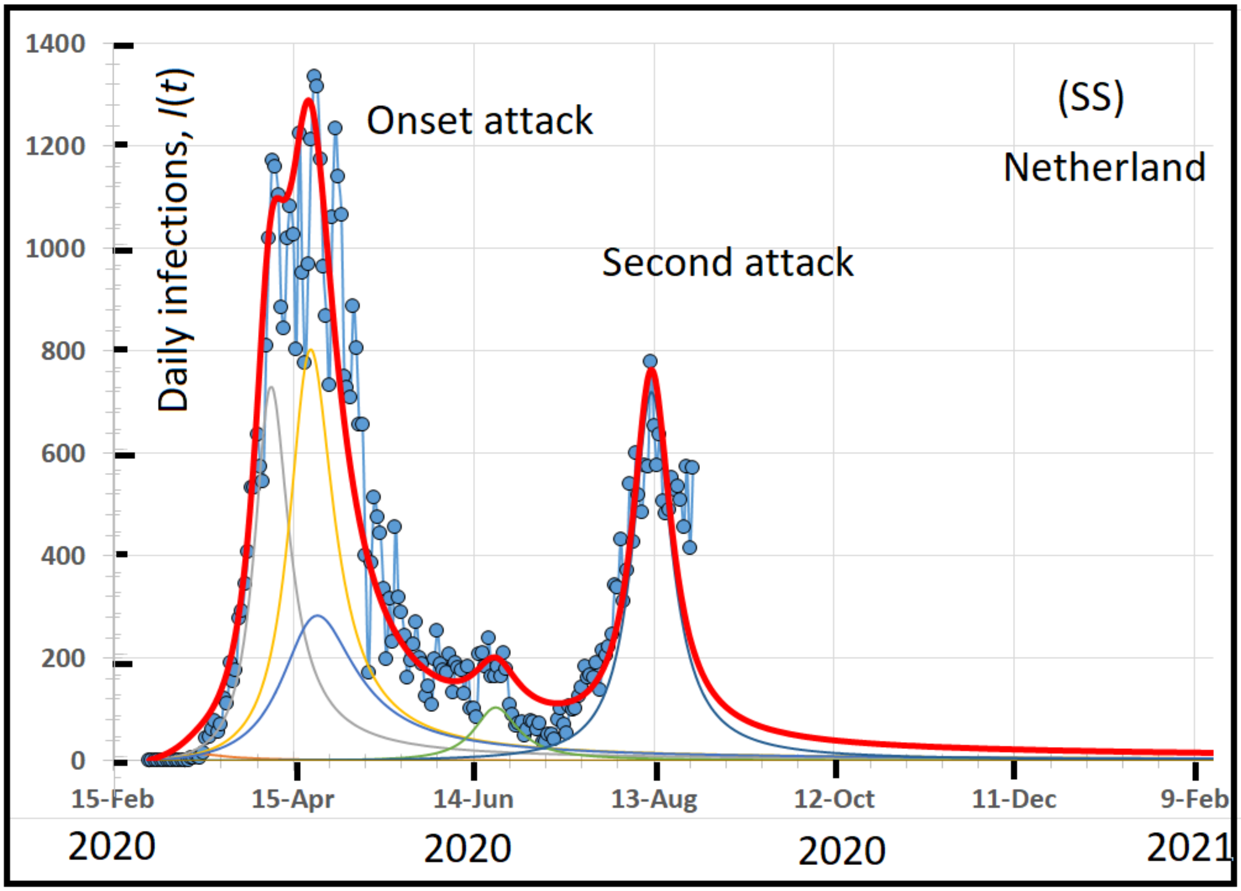

Fig. S19.

The daily infection of COVID 19 *I* (t) as a function of time, in Netherland. Blue circles are published data and continuous red line represents the resultant of five successive waves shown as fine coulored lines and calculated after equation (4). The onset attack has a calculated maximum of 1280 infections on April 21, 2020; the second attack had happened of 796 infections on August 12, 2020.

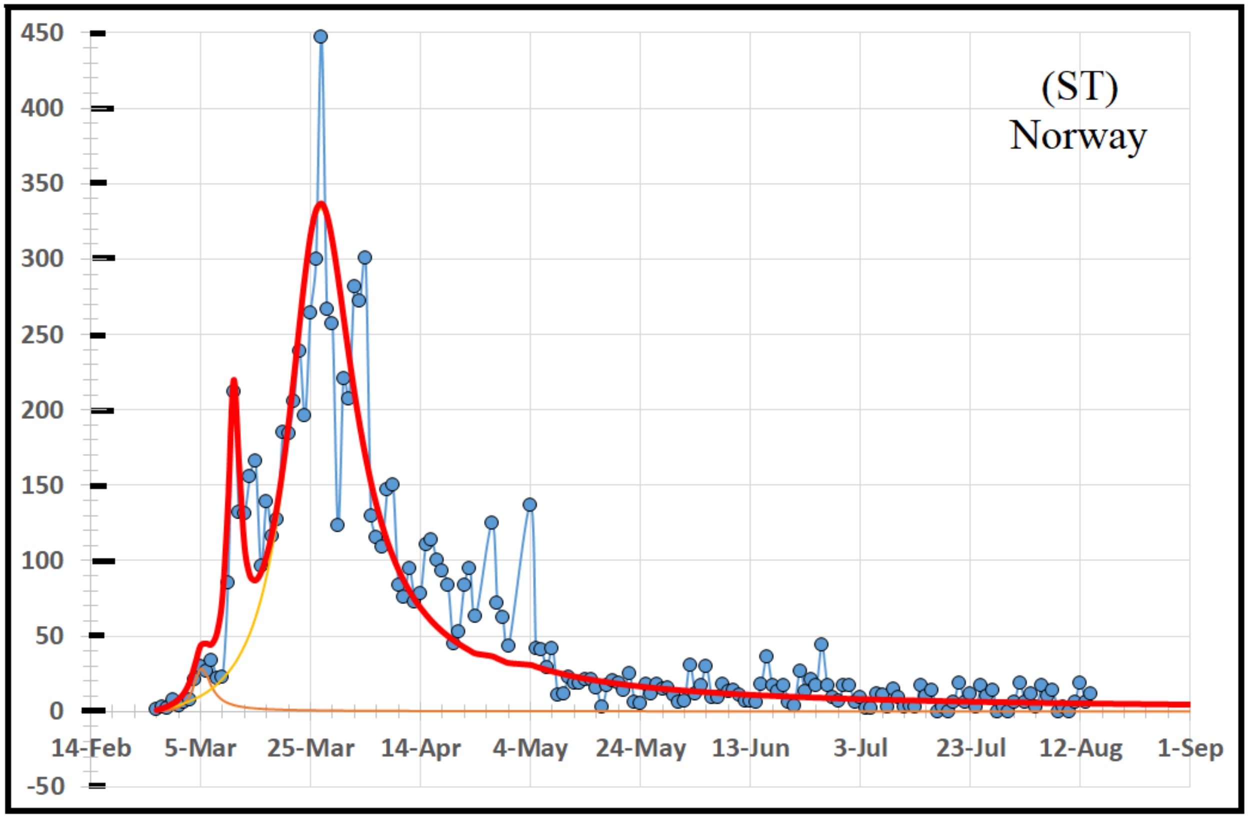

Fig. S19.

The daily infection of COVID 19 *I* (t) as a function of time, in Norway. Blue circles are published data and continuous red line represents the resultant of three successive waves shown as fine coulored lines and calculated after equation (4).

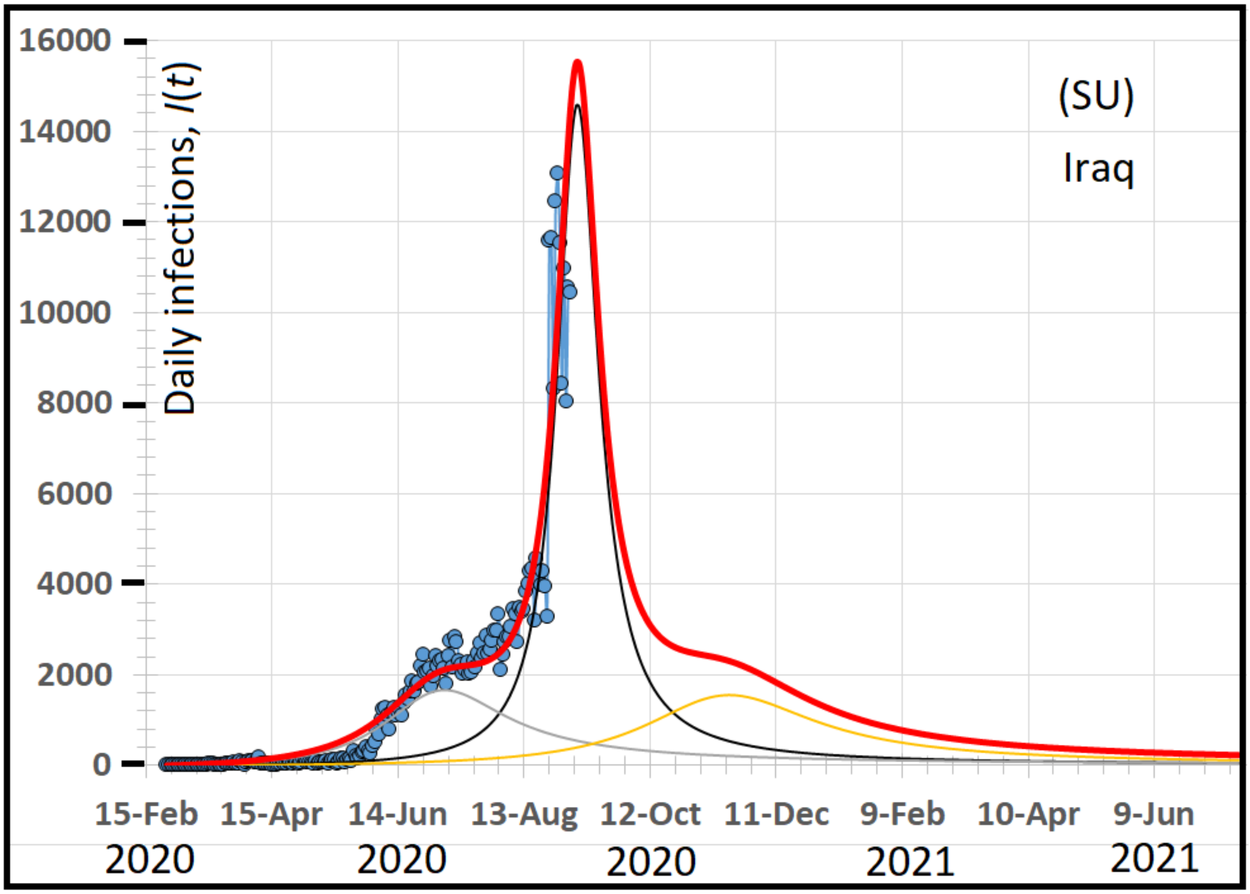

Fig. S20.

The daily infection of COVID 19 *I* (t) as a function of time, in Iraq. Blue circles are published data and continuous red line represents the resultant of three successive waves shown as fine coulored lines and calculated after equation (4).

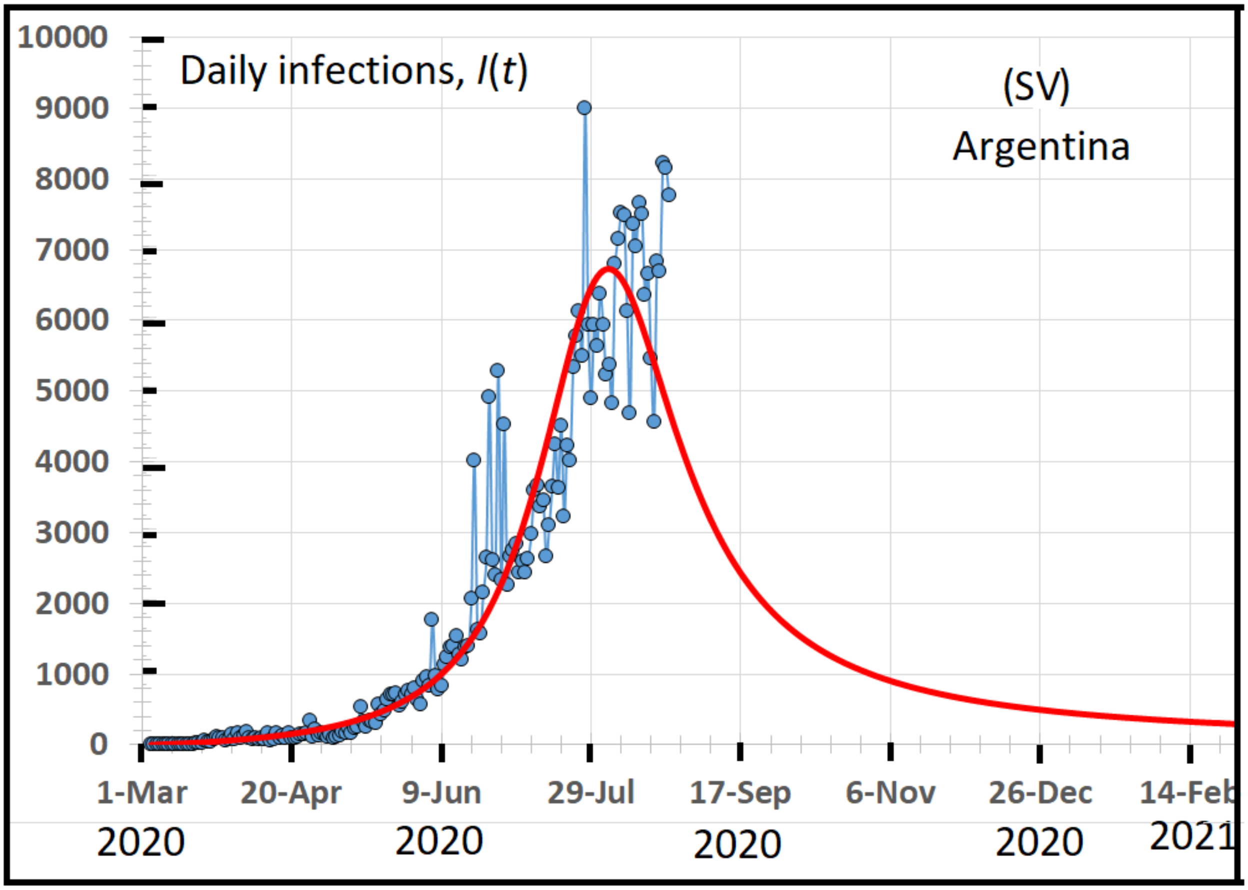

Fig. S21.

The daily infection of COVID 19 I (t) as a function of time, in Argentina. Blue circles are published data and continuous red line represents the resultant of one wave shown as fine coulored lines and calculated after equation (4).

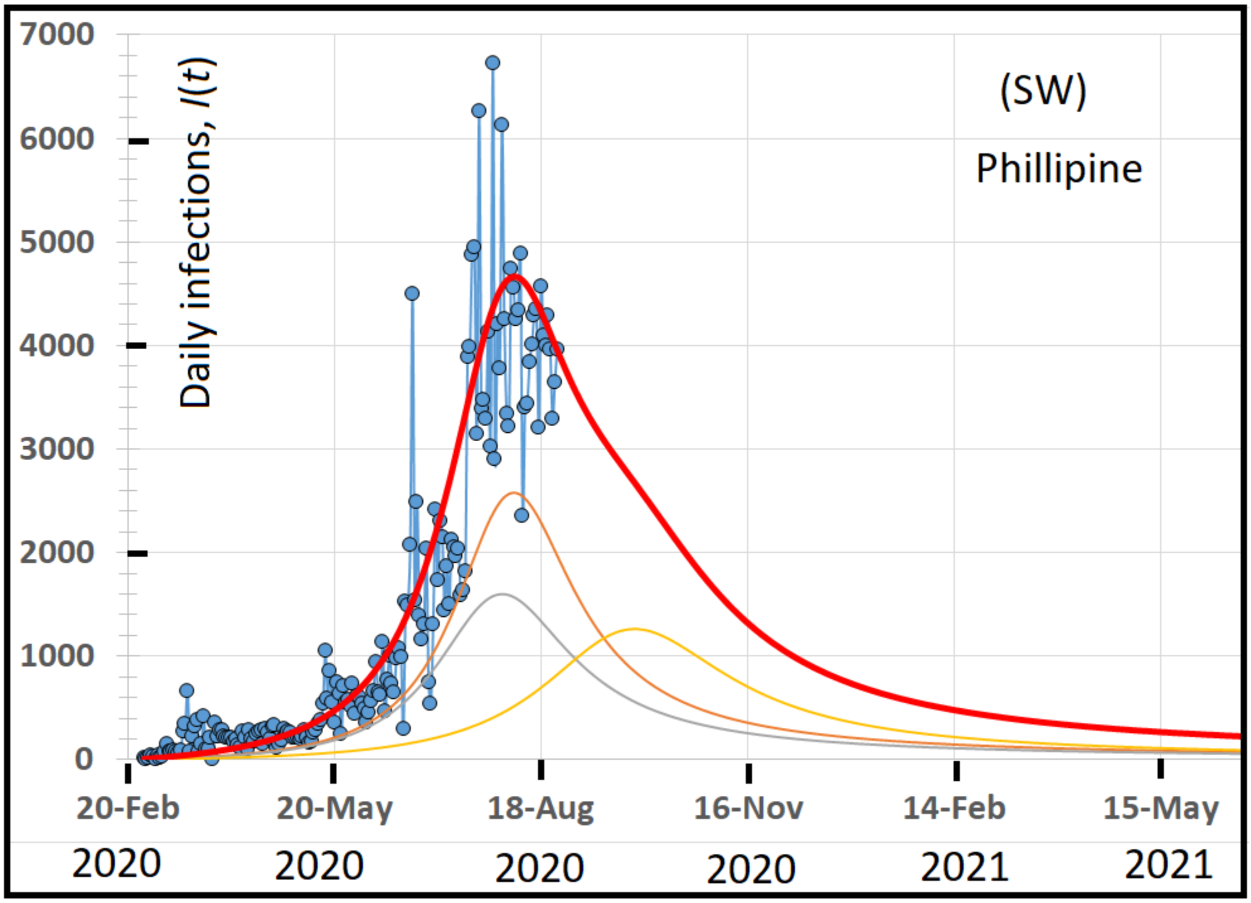

Fig. S22.

The daily infection of COVID 19 *I* (t) as a function of time, in Phillipine. Blue circles are published data and continuous red line represents the resultant of three successive waves shown as fine coulored lines and calculated after equation (4).

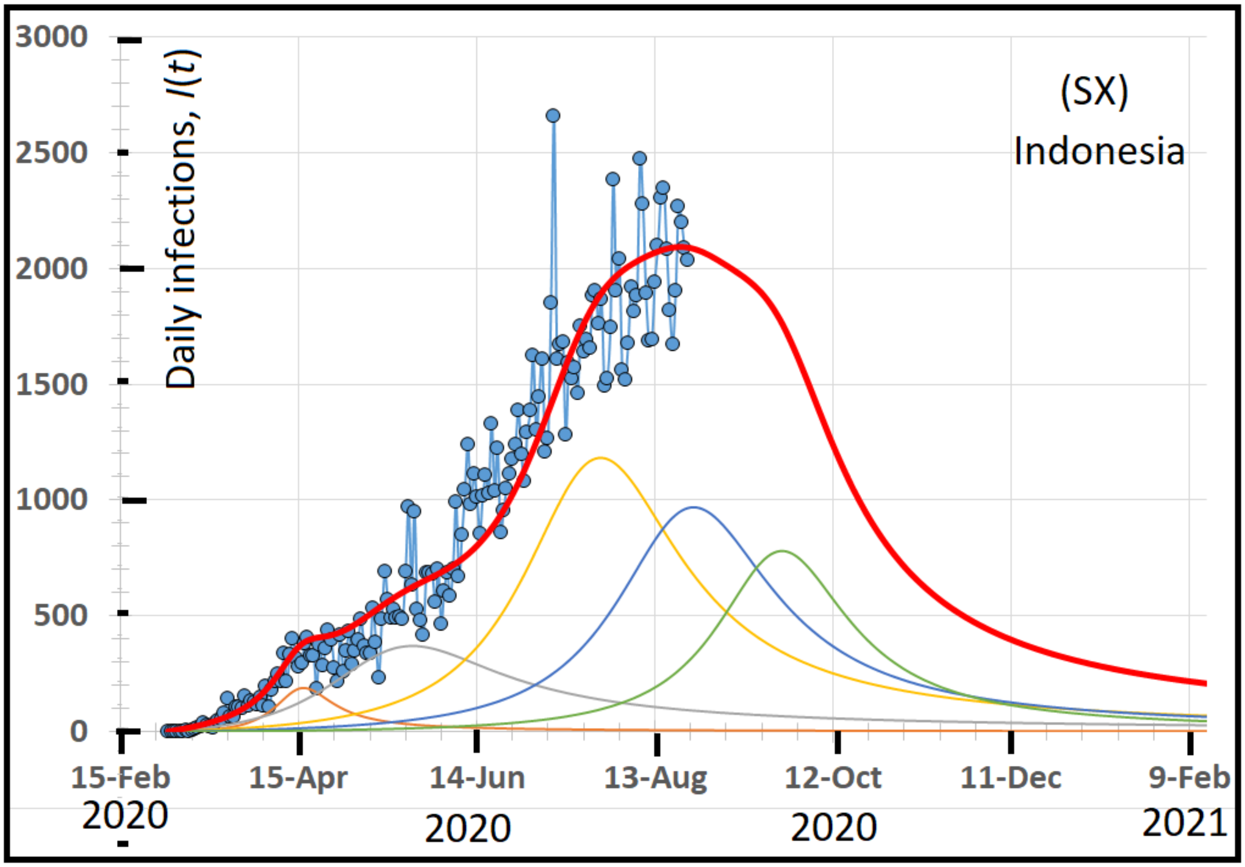

Fig. S23.

The daily infection of COVID 19 *I* (t) as a function of time, in Indonesia. Blue circles are published data and continuous red line represents the resultant of five successive waves shown as fine coulored lines and calculated after equation (4).

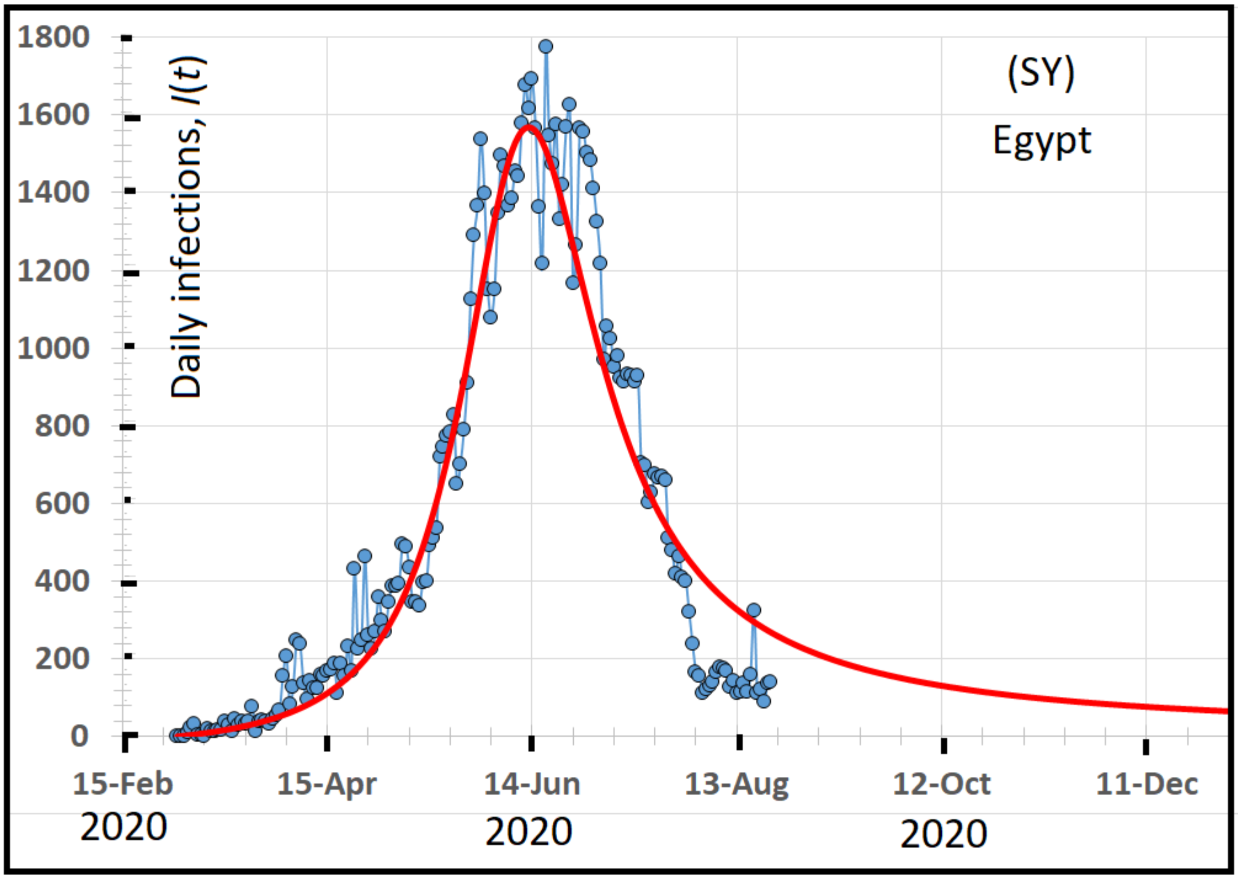

Fig. S24.

The daily infection of COVID 19 *I* (t) as a function of time, in Egypt. Blue circles are published data and continuous red line represents the resultant of three successive waves shown as fine coulored lines and calculated after equation (4).

Table S1

Country	W. No.	delay(δ)	B_0	$B(\infty)$	ω
USA	1	74	24	570	0.243902
USA	2	79	16	3988	0.057143
USA	3	102	12	2418	0.044843
USA	4	160	23	2740	0.019881
USA	5	170	35	789	0.083333
USA	6	181	17	1968	0.095238
USA	7	188	24	1241	0.03012
USA	8	211	17	3503	0.025126
USA	9	219	15	711	0.040486

Country	W. No.	delay(δ)	B_0	$B(\infty)$	ω
Brazil	1	118	22	4189	0·031546
Brazil	2	142	21	4607	0·031153
Brazil	3	166	17	3107	0·031447

Country	W. No.	delay(δ)	B_0	$B(\infty)$	ω
India	1	199	21	12665	0.027548

Country	W. No.	delay(δ)	B_0	$B(\infty)$	ω
Russia	1	81	19	198	0.188679
Russia	2	98	16	616	0.119048
Russia	3	108	17	607	0.063694
Russia	4	131	13	882	0.044643
Russia	5	169	13	787	0.026385
Russia	6	209	16	201	0.03125
Russia	7	269	15	613	0.03663

Country	W. No.	delay(δ)	B_0	$B(\infty)$	ω
S. Africa	1	141	15	232	0·05618
S. Africa	2	145	16	218	0·0625
S. Africa	3	154	17	528	0·05102
S. Africa	4	167	18	1185	0·034247
S. Africa	5	187	45	523	0·031153
S. Africa	6	244	17	219	0·027624

Country	W. No.	delay(δ)	B_0	$B(\infty)$	ω
Peru	1	85	22	341	0.046083
Peru	2	104	24	547	0.039526

Peru	3	107	12	159	0.27027
Peru	4	121	16	290	0.04329
Peru	5	175	13	543	0.03367
Peru	6	176	15	212	0.136986

Country	W. No.	delay(δ)	B_0	$B(\infty)$	ω
Mexico	1	102	12	468	0·04
Mexico	2	131	24	598	0·034483
Mexico	3	159	21	484	0·034483
Mexico	4	176	18	298	0·037453
Mexico	5	210	17	167	0·03367

Country	W. No.	delay(δ)	B_0	$B(\infty)$	ω
Chile	1	105	24	602	0·051546
Chile	2	123	15	567	0·051813

Country	W. No.	delay(δ)	B_0	$B(\infty)$	ω
Spain	1	42	53	1197	0·138889
Spain	2	54	24	411	0·088496
Spain	3	65	16	389	0·081301

Country	W. No.	delay(δ)	B_0	$B(\infty)$	ω
UK	1	48	24	520	0·052632
UK	2	56	53	186	0·138889
UK	3	65	35	491	0·083333
UK	4	80	16	473	0·066667
UK	5	110	18	47	0·111111

Country	W. No.	delay(δ)	B_0	$B(\infty)$	ω
Iran	1	21	16	156	0·357143
Iran	2	29	15	76	0·37037
Iran	3	44	35	515	0·125
Iran	4	59	17	176	0·071429
Iran	5	85	21	45	0·163934
Iran	6	95	12	107	0·123457
Iran	7	110	12	30	0·434783
Iran	8	123	15	298	0·039526
Iran	9	147	12	218	0·035714
Iran	10	179	12	187	0·03663

Country	W. No.	delay(δ)	B_0	$B(\infty)$	ω
Pakistan	1	85	15	238	0·053191

Pakistan	2	121	35	732	0·064516
Pakistan	3	143	14	61	0·043668

Country	W. No.	delay(δ)	B_0	$B(\infty)$	ω
S. Arabia	1	69	35	142	0·091743
S. Arabia	2	79	12	92	0·058824
S. Arabia	3	93	25	155	0·125
S. Arabia	4	123	15	172	0·120482
S. Arabia	5	128	17	285	0·034483
S. Arabia	6	135	15	41	0·144928
S. Arabia	7	143	16	286	0·046296

Country	W. No.	delay(δ)	B_0	$B(\infty)$	ω
Italy	1	59	53	415	0·07874
Italy	2	35	24	718	0·136986
Italy	3	41	16	971	0·088496

Country	W. No.	delay(δ)	B_0	$B(\infty)$	ω
Turkey	1	55	53	542	0·138889
Turkey	2	61	35	679	0·037037
Turkey	3	122	14	151	0·01996

Country	W. No.	delay(δ)	B_0	$B(\infty)$	ω
Colombia	1	128	18	164	0·035714
Colombia	2	148	15	393	0·035714
Colombia	3	162	16	386	0·078125
Colombia	4	196	15	166	0·041152
Colombia	5	228	17	103	0·044843

Country	W. No.	delay(δ)	B_0	$B(\infty)$	ω
Bangladesh	1	96	15	963	0·035088
Bangladesh	2	147	18	386	0·026316

Country	W. No.	delay(δ)	B_0	$B(\infty)$	ω
France	1	48	24	481	0·136986
France	2	49	16	674	0·090909
France	3	56	53	201	0·138889

Country	W. No.	delay(δ)	B_0	$B(\infty)$	ω
Canada	1	48	15	99	0·166667
Canada	2	65	18	328	0·057803
Canada	3	78	16	98	0·105263

Canada	4	98	15	75	0·089286

Country	W. No.	delay(δ)	B_0	$B(\infty)$	ω
Qatar	1	73	15	55	0·110497
Qatar	2	102	16	372	0·047619
Qatar	3	141	22	53	0·066667

Country	W. No.	delay(δ)	B_0	$B(\infty)$	ω
Belgium	1	46	49	251	0·081967
Belgium	2	54	15	167	0·079365
Belgium	3	56	32	112	0·065789

Country	W. No.	delay(δ)	B_0	$B(\infty)$	ω
Netherland	1	16	15	22	0·107527
Netherland	2	41	25	149	0·142857
Netherland	3	54	30	163	0·111111
Netherland	4	55	28	104	0·066667
Netherland	5	116	17	28	0·084034

Country	W. No.	delay(δ)	B_0	$B(\infty)$	ω
Philippine	1	158	15	376	0.027701
Philippine	2	164	19	519	0.031153
Philippine	3	214	17	307	0.02008
Philippine	4	215	15	224	0.030675

Country	W. No.	delay(δ)	B_0	$B(\infty)$	ω
Germany	1	61	16	115	0·47619
Germany	2	68	16	260	0·3125
Germany	3	76	21	315	0·232558
Germany	4	79	20	319	0·051813
Germany	5	158	13	27	0·151515
Germany	6	228	17	414	0·103093

Country	W. No.	delay(δ)	B_0	$B(\infty)$	ω
Norway	1	9	15	21	0·526316
Norway	2	15	13	29	0·769231
Norway	3	30	16	95	0·138889

Country	W. No.	delay(δ)	B_0	$B(\infty)$	ω
Iraq	1	128	15	476	0·027701
Iraq	2	196	19	719	0·082645
Iraq	3	214	15	224	0·030675

Iraq	4	264	16	307	0·02008

Country	W. No.	delay(δ)	B_0	B(∞)	ω
Indonesia	1	45	19	69	0·082645
Indonesia	2	75	17	186	0·027701
Indonesia	3	143	18	285	0·030581
Indonesia	4	175	15	194	0·030675
Indonesia	5	206	16	119	0·03663

Country	W. No.	delay(δ)	B_0	B(∞)	ω
Egypt	1	101	18	396	0.040486

Table S1.

Fitting parameters that fit published data to equation (4). The number of waves, n, varies in the range $1 < n < 11$. δ is the time delay for each wave. B(0) and B(∞) are the number of confined individuals at initial and infinite times, respectively. ω is the wave frequency given by equation (S6).

Calculated data are available under request from corresponding author

Evidence before this study

On August 22, 2020, we know better about severe acute respiratory syndrome coronavirus 2 (SARS-CoV-2) and our understanding are more accelerated to know more about tests, which has led to better way to impede the virus. We have used (R) package: medrxivr, and have searched the medRxiv preprint and PubMed. With the terms COVID 19; complex numbers; confinement; mathematical model; reproduction number, on August 22, 2020, our search have returned 173 points: 17 published papers and 156 preprints. Within these preprints, we have identified 19 papers deals with using mathematical models to estimate the role of testing in COVID-19 control and we identified seventy eight papers concerning the impact of confinement on the viral prolongation. Twelve publications have studied the confinement effects in their countries. Twenty three publications have modified the susceptible, exposed, infectious, and recovered SEIR model to account about the confinement and Lockdown of COVID-19 effects in different countries. Two publications have studied the modes of transmission of COVID-19 outbreak. One paper has investigated the nonsuccess of lockdown in England. One paper has discussed the SEIR model in view of random walk SIR-model. However, the estimated data are often divergent, reflecting different assumptions about the viral-epidemiology and testing capacity.

Added value of this study

The present work considers the total population as a complex number. Its imaginary umber equals the infected individuals, while the real part is proportional to the confined individuals. This has led to know that the virus propagates in successive waves, or in one only wave. One cannot find these ideas in the present literature. The model sheds the light on the importance of the individual's reaction to the first wave attack, which permits passing another waves or not. We combined the available evidences for the sensitivity and specificity of the existing tests along with the epidemiological data on the incidence of asymptomatic infection and the contribution of infected individuals without symptoms or symptoms to transmission. We conducted sensitivity analyzes to determine the robustness of our results and examined the implications of our results for national testing policies.

Implications of all the available evidence

Although of its economic recession, confinement is an essential parameter in the prevention of COVID-19 transmission, in addition to its established use for pandemic surveillance and confirmation of a COVID-19 diagnosis. However, scientists should

pay more efforts to get an optimal scenario between economy loss and health gain, in addition, efforts should be paid, also, to clarify the how to reproduction number get down than one.
